# Bone-Seeking Matrix Metalloproteinase Inhibitors for the Treatment of Skeletal Malignancy

**DOI:** 10.3390/ph13060113

**Published:** 2020-06-01

**Authors:** Antonio Laghezza, Luca Piemontese, Leonardo Brunetti, Alessia Caradonna, Mariangela Agamennone, Antonella Di Pizio, Giorgio Pochetti, Roberta Montanari, Davide Capelli, Marilena Tauro, Fulvio Loiodice, Paolo Tortorella

**Affiliations:** 1 Department of Pharmacy and Pharmaceutical Sciences, University of Bari “A. Moro”, via E. Orabona 4, 70125 Bari, Italy; antonio.laghezza@uniba.it (A.L.); luca.piemontese@uniba.it (L.P.); l.brunetti2@studenti.uniba.it (L.B.); a.caradonna@hotmail.it (A.C.); 2 Department of Pharmacy, University “G. d’Annunzio” of Chieti-Pescara, Via Dei Vestini, 31, 66100 Chieti, Italy; magamennone@unich.it; 3 Leibniz-Institute for Food Systems Biology at the Technical University of Munich, Lise-Meitner-Str. 34, 85354 Freising, Germany; antonelladipizio@gmail.com; 4 Istituto di Cristallografia, Consiglio Nazionale delle Ricerche, Montelibretti, Monterotondo Stazione, 00015 Roma, Italy; giorgio.pochetti@ic.cnr.it (G.P.); roberta.montanari@ic.cnr.it (R.M.); davide.capelli@ic.cnr.it (D.C.); 5 Tumor Biology Department, H. Lee Moffitt Cancer Center and Research Institute, Tampa, FL 33612, USA; marilena.tauro@moffitt.org

**Keywords:** bisphosphonates, matrix metalloproteinase inhibitors, antitumor agent, bone targeting, skeletal malignancies

## Abstract

Matrix metalloproteinases (MMPs) are a family of enzymes involved at different stages of cancer progression and metastasis. We previously identified a novel class of bisphosphonic inhibitors, selective for MMPs crucial for bone remodeling, such as MMP-2. Due to the increasing relevance of specific MMPs at various stages of tumor malignancy, we focused on improving potency towards certain isoforms. Here, we tackled MMP-9 because of its confirmed role in tumor invasion, metastasis, angiogenesis, and immuno-response, making it an ideal target for cancer therapy. Using a computational analysis, we designed and characterized potent MMP-2/MMP-9 inhibitors. This is a promising approach to develop and clinically translate inhibitors that could be used in combination with standard care therapy for the treatment of skeletal malignancies.

## 1. Introduction

Matrix metalloproteinases (MMPs) are a family of 23 enzymes that control extracellular matrix (ECM) remodeling and act as key regulators of cancer-bone interactions in skeletal malignancy [1]. Numerous functions have been assigned to these proteins in bone tissue, including osteoblast/osteocyte differentiation, bone formation, solubilization of the osteoid during bone resorption, osteoclast recruitment and migration, and as a coupling factor in bone remodeling under physiological conditions. In turn, several pathologies, associated with imbalances in bone remodeling, arise mainly from MMP overexpression and abnormalities of the ECM, leading to pathologic bone osteolysis or bone formation [2].

An imbalance of the MMP enzymatic activity/endogenous inhibitor ratio leads to irregular bone remodeling and tissue destruction. These processes can occur under inflammatory conditions (periodontitis and rheumatoid arthritis) [3], in metabolic disorders (osteoporosis) [4], human genetic mutations, bone tumors, and bone metastases. Bone metastases represent a common event in tumor progression present in more than 80% of patients with advanced breast or prostate cancer and in approximately 15–30% of patients with carcinoma of the thyroid, lung, bladder, or kidney [5,6]. In addition, melanoma and multiple myeloma also readily metastasize to the skeleton [7]. Once tumors metastasize to bone, the disease becomes incurable, and patients may experience several skeletal-related complications, such as severe bone pain, hypercalcemia, nerve compression syndromes, and pathological fractures. This severely increases morbidity and diminishes the quality of life of the patients [7,8,9].

Tumor–bone interaction has been classically described as a “vicious cycle” in which cancer cells promote the expression of potent osteoclastogenic factors such as RANK ligand by bone-lining osteoblasts [1,8]. This leads to the formation of multinucleated osteoclasts that, in turn, resorb the mineralized matrix and release growth factors, which then drive cancer cell growth [10]. Several studies have shown that MMPs are key regulators of this “vicious cycle” [1] and provide a rationale for the application of MMP inhibitors (MMPIs) for the treatment of bone metastatic disease. However, enthusiasm for MMPIs in the clinical setting has been dampened by the results of unsuccessful clinical trials performed with broad-spectrum inhibitors over the past two decades [11]. The reasons for these failures are several, including dose-limiting toxicity and the lack of knowledge pertaining to non-matrix MMP substrates [12]. Indeed, both the systemic expression of these enzymes and the large number of pathways in which MMP is involved imply that off-target effects and dose-limiting toxicities would be a serious concern [13]. For these reasons, structural characterization of the mechanisms of action of these enzymes is fundamental to target their uncontrolled actions [14,15].

Another factor that negatively influenced the development of MMPIs is the presence of the hydroxamic function in the clinically studied compounds. This functional group, in fact, is a good zinc binding group (ZBG) but possesses a detrimental effect on selectivity and pharmacokinetics. For this reason, new scaffolds with alternative ZBGs have been developed—for example, potent MMPIs were obtained using phosphonate [16], phosphinic, and phosphinate functional groups to reproduce the gem-diol intermediate of the transition state during peptide hydrolysis [17].

In recent papers, we have shown that the use of bisphosphonic-based bone-seeking MMP inhibitors (BMMPIs) makes the specific targeting of individual MMPs in the bone microenvironment a feasible strategy [18,19]. The compounds we developed showed particular selectivity towards MMP-2 and a very interesting activity against carbonic anhydrases involved in bone remodeling [20,21]. 

**Figure 1:**
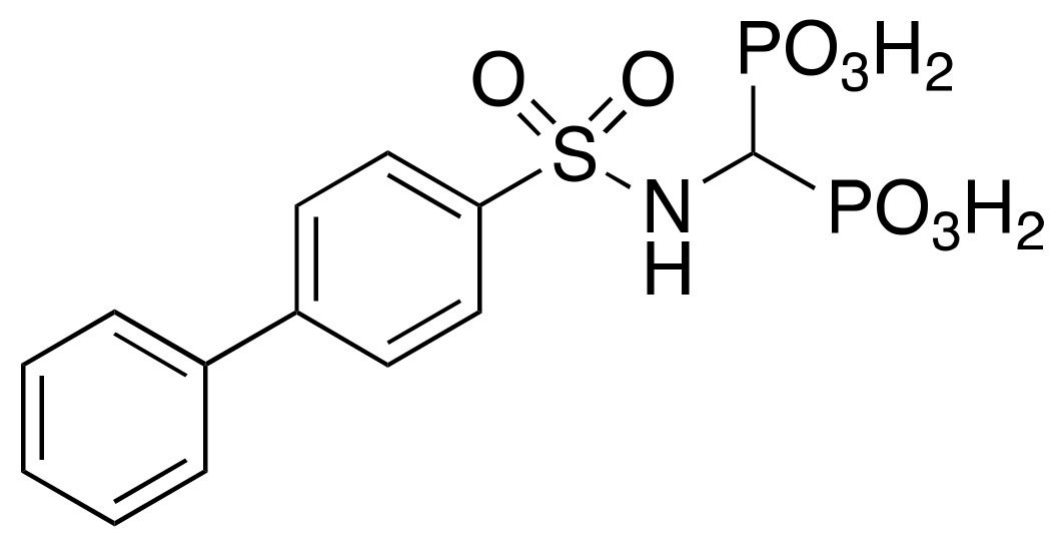
Chemical structure of inhibitor ML 115.

Among BMMPIs, ML 115 (Figure 1) was the most potent and specific and also showed antiosteoclastic activity. This particular activity profile would allow for a reduction in skeletal morbidity in patients with lytic bone disease.

These novel bone-seeking MMPIs demonstrated that metastatic breast cancer growth in bone and cancer-inducer osteolysis in multiple myeloma can be reduced in vivo [22,23]. Furthermore, the BMMPIs are well-tolerated, with no side effects noted, even at the chosen dose of 25 mg/kg three times per week over the course of four weeks. In addition, BMMPIs are superior in promoting tumor apoptosis compared with the standard-of-care bisphosphonate, zoledronate [23].

Although the inhibition of MMP-2 is undoubtedly beneficial for the reduction of tumor burden and for the prevention and treatment of metastases [24], a useful therapeutic strategy could be to target other individual MMPs that play a role in the progression of metastatic cancers. One such target is MMP-9, which has been shown to control angiogenesis in multiple myeloma [25] and is capable of driving the formation of metastatic niches in breast cancer [26]. Moreover, genetic ablation of MMP-9 results in a reduction in a myeloma murine model [27]. Therefore, in this paper, we report the design and synthesis of BMMPIs capable of inhibiting both MMP-2 and MMP-9, since a dual inhibition of both MMP isoforms could enhance their therapeutic potential. 

## 2. Results and Discussion

In order to obtain detailed structural information on the binding mode of the bisphosphonate group, we tried to acquire the crystal structure of MMP-9 in a complex with the inhibitor ML 115. However, the crystallization trials did not give the expected results; therefore, we decided to solve the crystal structure of the complex between that inhibitor and MMP-8, given the well-established protocols of purification and crystallization of the catalytic domain of this particular MMP isoform [28,29,30] and the high similarity of the catalytic site of the two enzymes (statistics of crystallographic data and refinement are reported in Table S1). The most relevant difference between them resides in the number and type of residues of the omega-loop (13 for MMP-8 with respect to 11 of MMP-9) that determines differences in the bottom of the S1’ pocket. This difference can influence the specificity of ligands that deeply occupy this pocket.

**Figure 2:**
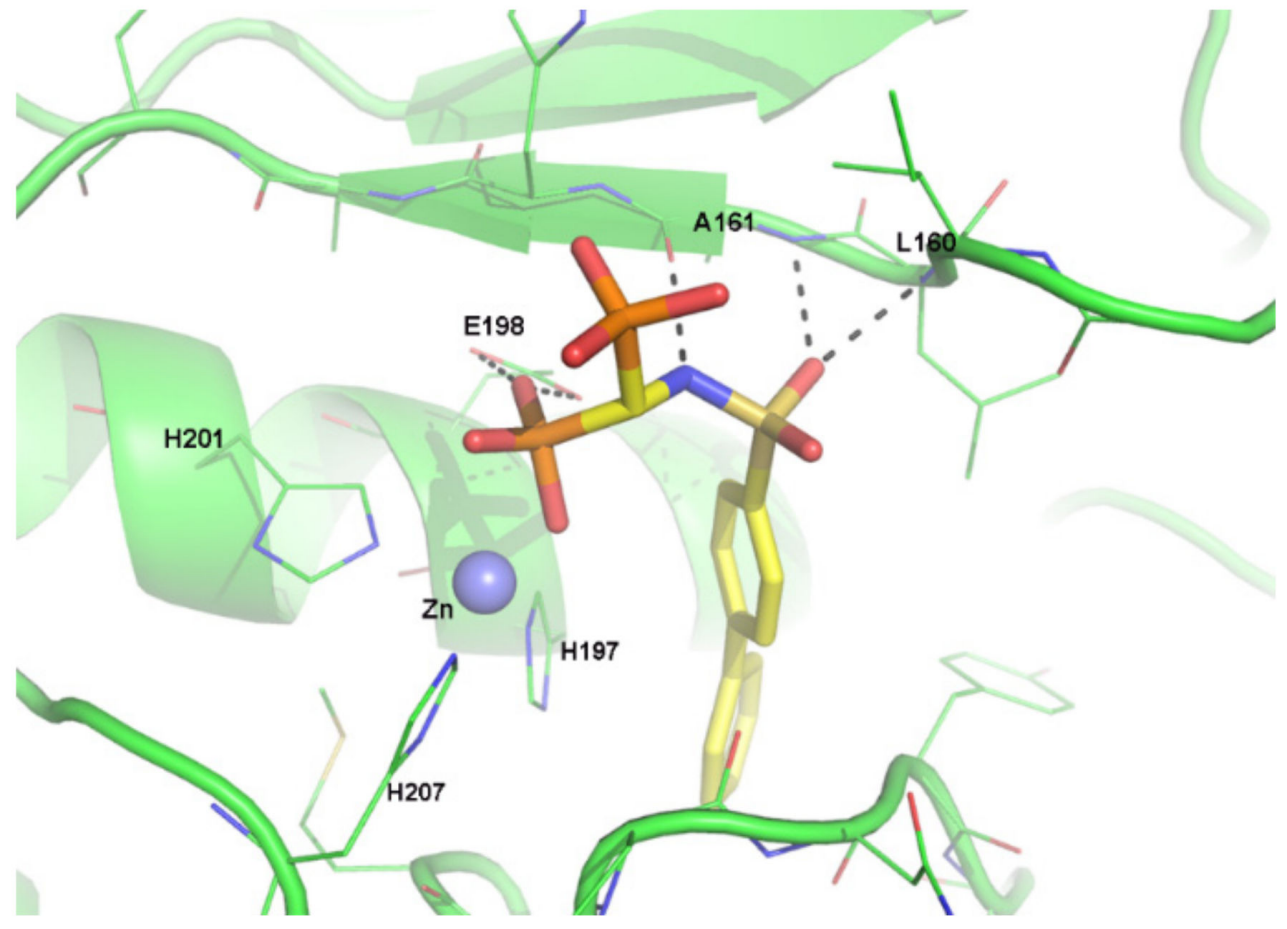
X-ray crystal structure of the ML 115 in complex with matrix metalloproteinase (MMP)–8. Hydrogen bonds between ML 115 and MMP–8 are shown as grey, dashed lines. The zinc ion is represented as a violet sphere. (PDB CODE: 4QKZ).

ML 115 binds the catalytic zinc ion of MMP-8 in a monodentate fashion with one phosphonate moiety (Figure 2, Figure S1). Meanwhile, the second phosphonate group is oriented towards the solvent. One oxygen of the first phosphonate moiety is also hydrogen-bonded with the catalytic Glu198 side chain. The sulfonamide moiety is hydrogen-bonded with the backbone NH of Leu160 and the backbone NH and CO of Ala161 belonging to the antiparallel β-strand of MMP–8. As expected, the biphenyl group is inserted into the S1’ site. Extended hydrophobic interactions with the S1’ loop account for high MMPI activity. 

**Figure 3:**
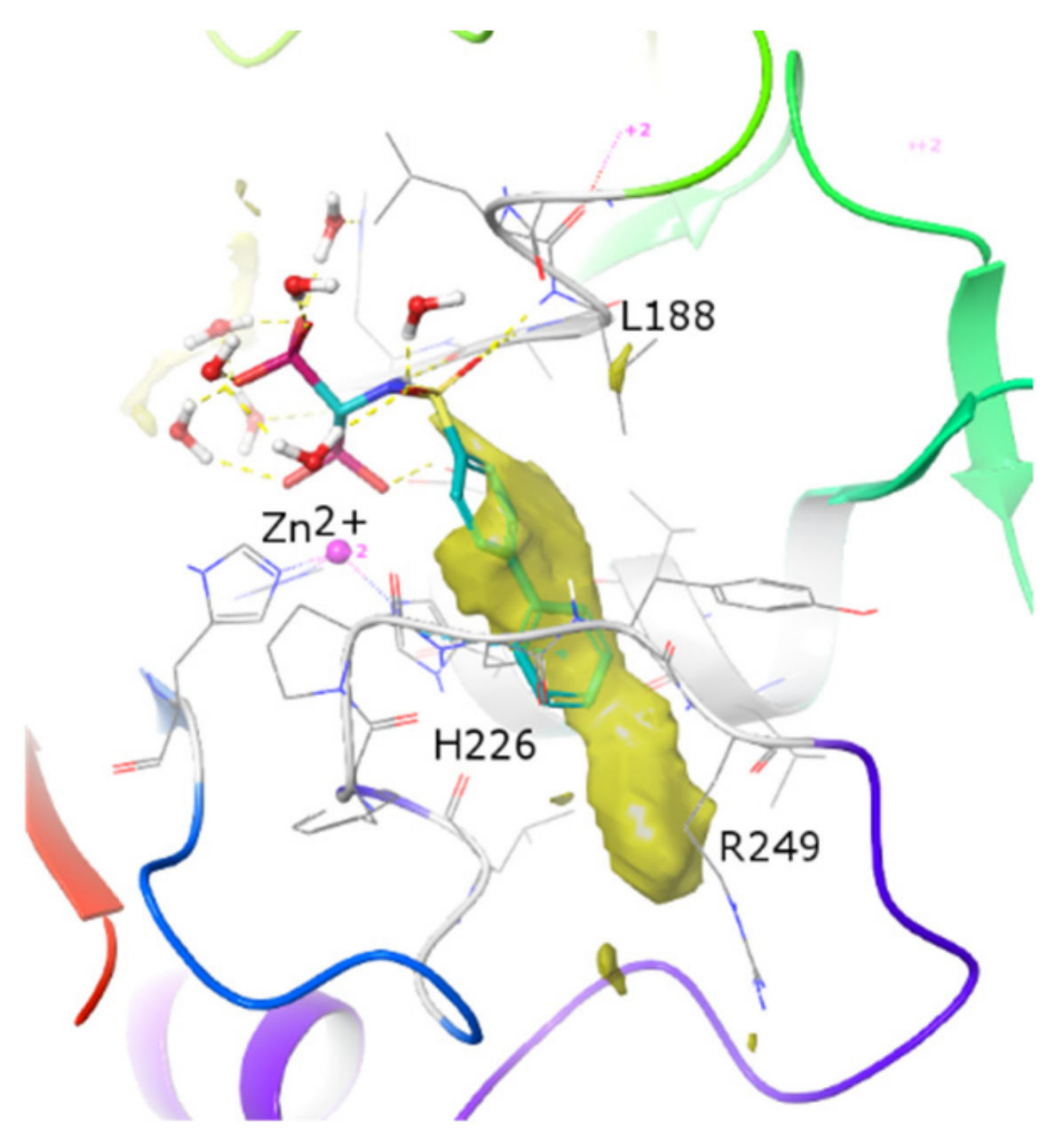
The molecular interaction field for the hydrophobic probe (yellow transparent surface) calculated by SiteMap [31] using default parameters in the MMP-9 S1’ site (PDB ID: 4H3X) where ML 115 has been docked, maintaining the experimental coordination geometry of the bisphosphonic group.

The obtained binding pose overlaps very well with a prior potent phosphonic sulfonamide with nanomolar potency towards MMPs [28]. The comparison of the two ligands’ geometries (Figure S2, Supplementary Materials) explains the reduced activity of BMMPIs with respect to monophosphonates: in fact, while the first phosphonate forms the same monodentate bond with the zinc ion, the second phosphonic group of ML 115 overlaps with the isopropyl side chain of the monophosphonic ligand by substituting a hydrophobic group, which positively interacts with the Ile159 (MMP-8 numbering) side chain in the S1 site, with an ionic group. However, the presence of the bisphosphonic function is mandatory to target the bone tissue; therefore, the design of more active ligands—in particular, towards MMP-9—was tackled by the modification of the aromatic portion exploring the S1’ site. To this aim, we docked ML 115 into the MMP-9 binding site to hypothesize the structural modifications that could improve the inhibition of this isoform. Moreover, to better depict the S1’ site, a SiteMap analysis was carried out on the MMP-9 binding site (Figure 3) that shows a partial occupancy of the extended hydrophobic field in the MMP-9 S1’ site by ML 115. 

**Figure 4:**
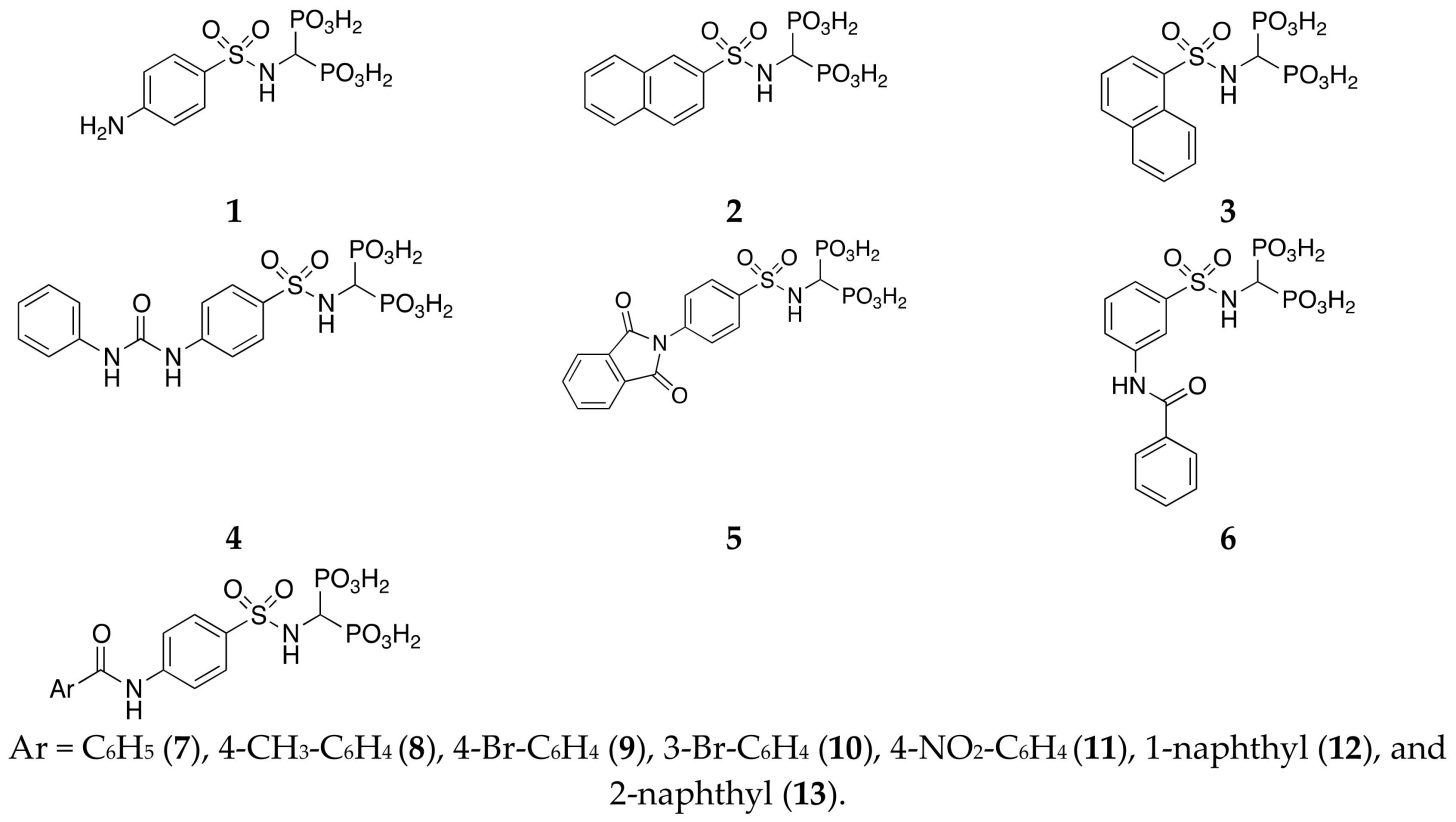
Chemical structures of compounds **1–13**.

This evidence prompted us to design a new series of BMMPIs (Figure 4), most of which are characterized by an elongation of the aromatic portion of ML 115 to exploit further interactions in the S1’ site. To this end, the diphenyl residue was substituted with a naphthalene moiety (compounds **2–3**) or a spacer was inserted between the two phenyl rings (compounds **4–13**) by synthesizing compound **1** as an intermediate.

**Scheme 1:**
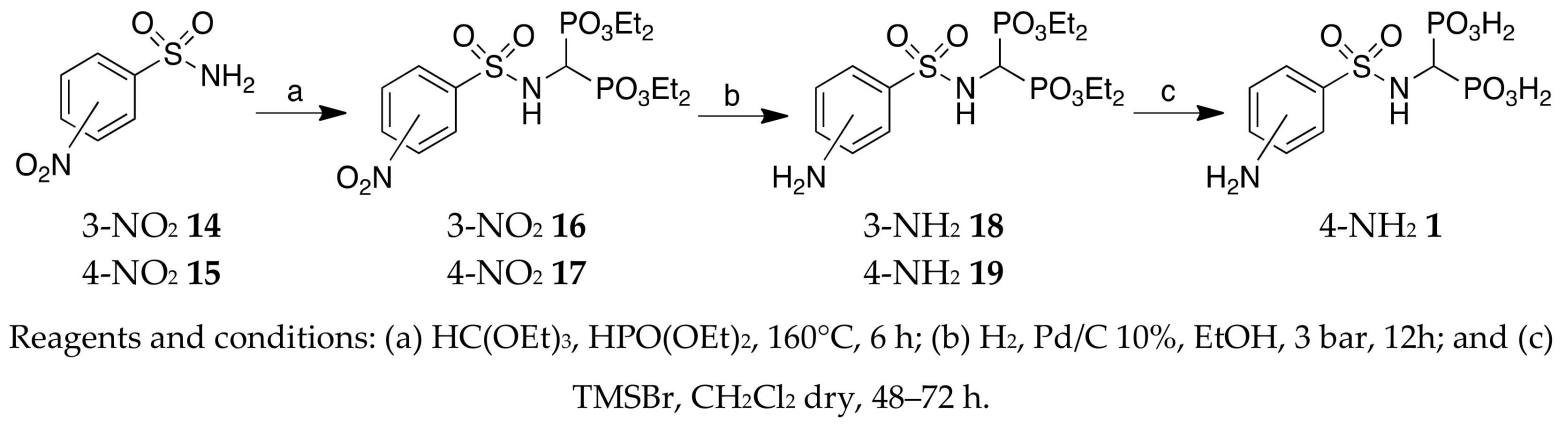
Synthesis of compound **1**.

**Scheme 2:**
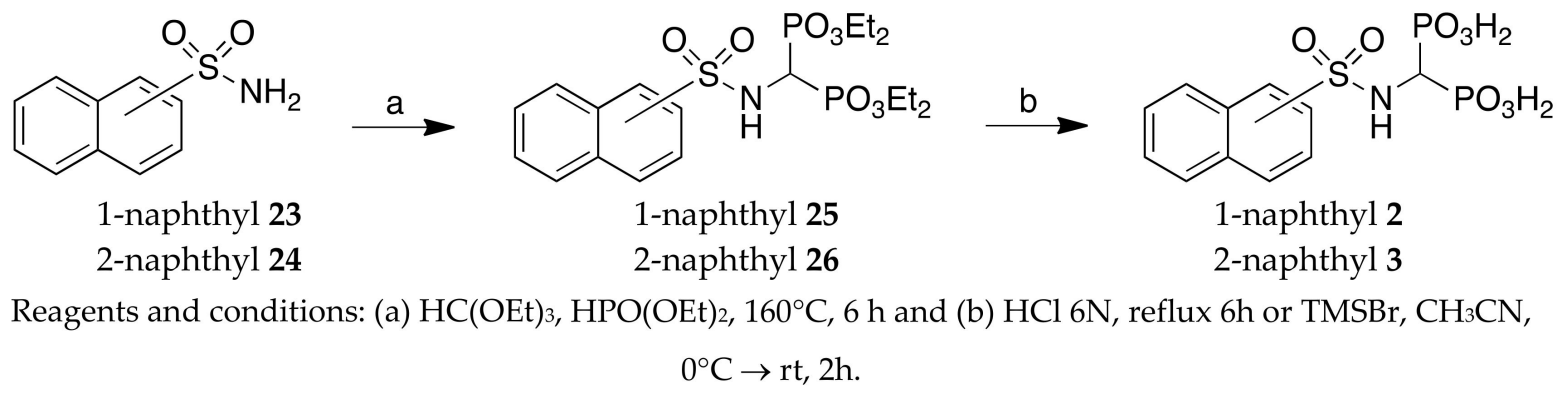
Synthesis of compounds **2** and **3**.

Bisphosphonic acids **1–13** were prepared by treating the appropriate commercially available sulfonyl chloride with 30% NH_4_OH to obtain the corresponding sulfonamides **14–15** (Scheme 1) and **23–24** (Scheme 2). Resulting compounds were treated with triethylorthoformate and diethyl phosphite to give the tetraethyl-1,1-bisphosphonates **16–17** and **25–26**. Reduction of the nitro group of tetraethyl (4-nitro-phenylsulfonylamino)methyl-1,1-bisphosphonate (**17**) and dealkylation of bisphosphonate esters **19**, **25,** and **26** under acidic conditions or with TMSBr afforded the desired bisphosphonic acids **1–3**. 

**Scheme 3:**
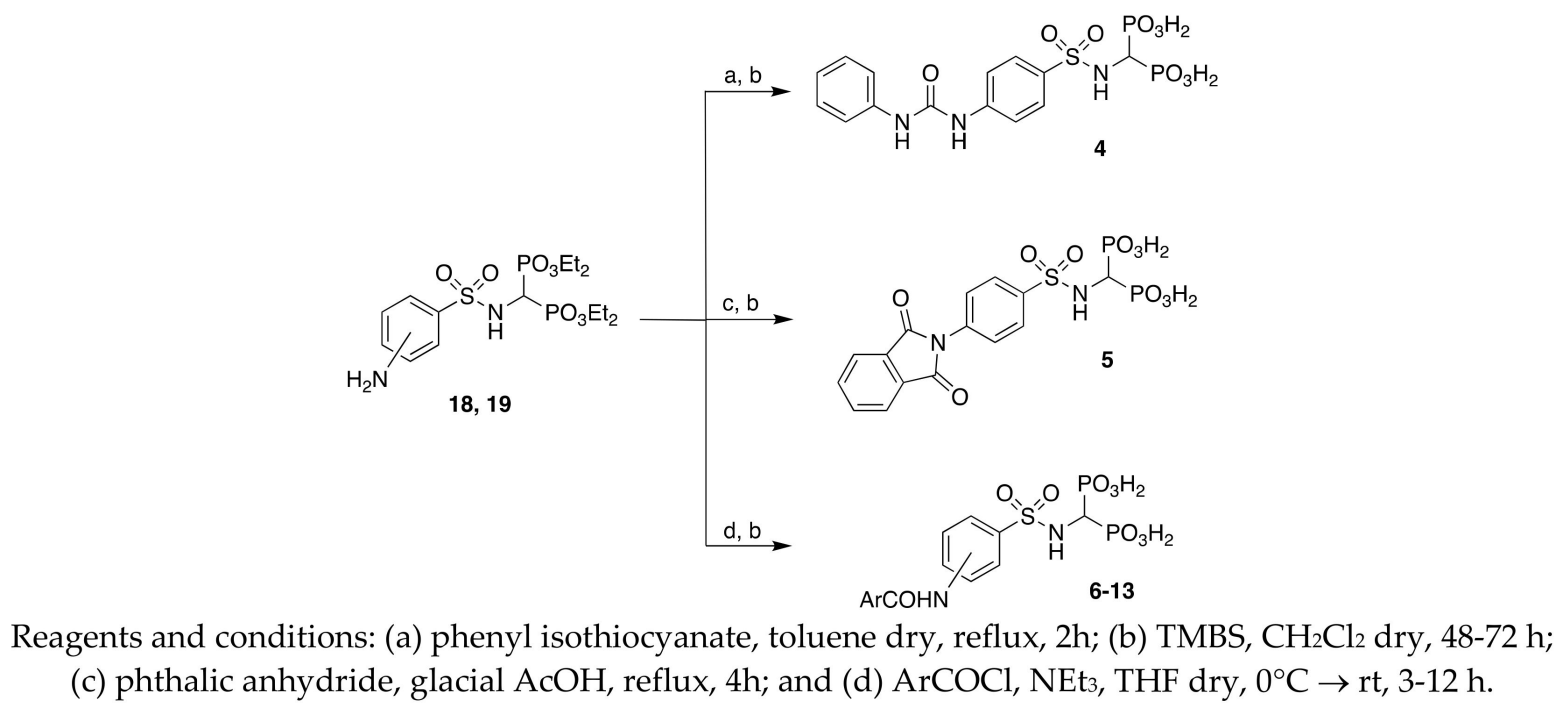
Synthesis of compounds **4–13**.

The synthesis of bisphosphonic acids **4–13** (Scheme 3) involves the acylation of the anilino derivatives **18** and **19** with phenyl isothiocyanate, phthalic anhydride, or the appropriate acyl chloride. Dealkylation of bisphosphonate esters with TMSBr afforded bisphosphonic acids **4–13**.

All synthesized derivatives **1–13** were tested against MMP-2, -8, -9, and -13 (Table 1) and compared with ML 115 as the reference compound.

The presence of a 4-amino group (**1**) led to a loss of potency towards all tested MMPs of approximately one order of magnitude compared to the reference compound; however, relative selectivity towards MMP-2 was kept.

The substitution of biphenyl moiety of ML 115 with 1- or 2-naphthyl (**2** and **3**) resulted in a decrease of activity towards MMP-2 and -8, as expected for the long and narrow S1’ pocket of these two isozymes, although compound **3** showed a micromolar activity towards all tested MMPs without any particular selectivity. 

The introduction of a 3-phenylureido moiety (**4**) or phthalic group (**5**) as side chain led to compounds with improved activity towards MMP-9, while retaining a five-times higher potency towards MMP-2. Compounds **4** and **5** showed similar activity profiles towards MMP-9 and -13, while being less potent towards MMP-8.

Compound **6** can be seen as a less rigid analogue of compound **5**; its additional flexibility led to a higher overall potency towards all tested MMPs (in the low micromolar range), while eliminating any kind of selectivity. The corresponding isomer **7**, however, showed a significant loss of potency, with evident selectivity towards MMP-2. 

The substitution of the aromatic ring of the benzamide side chain of compound **6** was then explored. Different groups (methyl, bromine, and nitro groups) were evaluated, obtaining new compounds with interesting inhibition profiles. All substitutions in 4’-position (mainly compounds **9** and **11**) led to a significant increase in activity towards MMP-9. Compound **9**, in particular, showed the best activity profile in terms of potency (in the nanomolar range) and selectivity towards MMP-9, while maintaining a good activity for the other isoforms. 

Compound **10**, bearing a bromine atom in 3’ position, showed a complete loss of activity on MMP-9 and, overall, an inferior activity profile towards all tested MMPs when compared to its isomer compound **9**. 

The introduction of bulkier substituents, as seen for compounds **12** and **13**, did not translate in higher activity towards MMP-9. Indeed, these compounds showed a certain selectivity towards MMP-2, with potency in the low micromolar range.

To investigate the BMMPI-binding mechanism, molecular-docking studies were performed on the MMP-2, MMP-8, MMP-9, and MMP-13 catalytic sites. 3D structures used for this study were selected through crossdocking studies, as previously reported [32]. Water molecules surrounding the bisphosphonic function in the experimental structures were maintained in the docking simulations in order to allow for water-mediated interactions of the zinc-binding group with the protein [33].

Docking calculations were carried out using Glide [31], applying both standard precision (SP) and extra precision (XP) protocols, calculating the final score with the Molecular Mechanics—Generalized Born (MM-GBSA) method. The obtained scores showed a good ability to distinguish between active and inactive compounds (enrichment values are reported in the Supplementary Materials, Table S2 and Figure S3).

Notably, docking calculations carried out on the original MMP-8:ML115 complex (4KQZ) were not successful in justifying the activity of ligands with longer aromatic moieties. Indeed, MMP-8 S1’ site is hindered by the Arg222 side chain that is involved in the conformational change of the S1’ loop, leading to the disclosure of the S1’* site in the MMP-8 [34]. Therefore, to rationalize the activity of larger compounds (8, 9, and 11), the docking calculations were carried out using the X-ray geometry of MMP-8 in a complex with a ligand binding an extra region at the bottom of the S1’ site [34]. On the other hand, flexibility is an important general issue in ligand-target interactions and is particularly relevant for MMPs [35]. 

**Figure 5:**
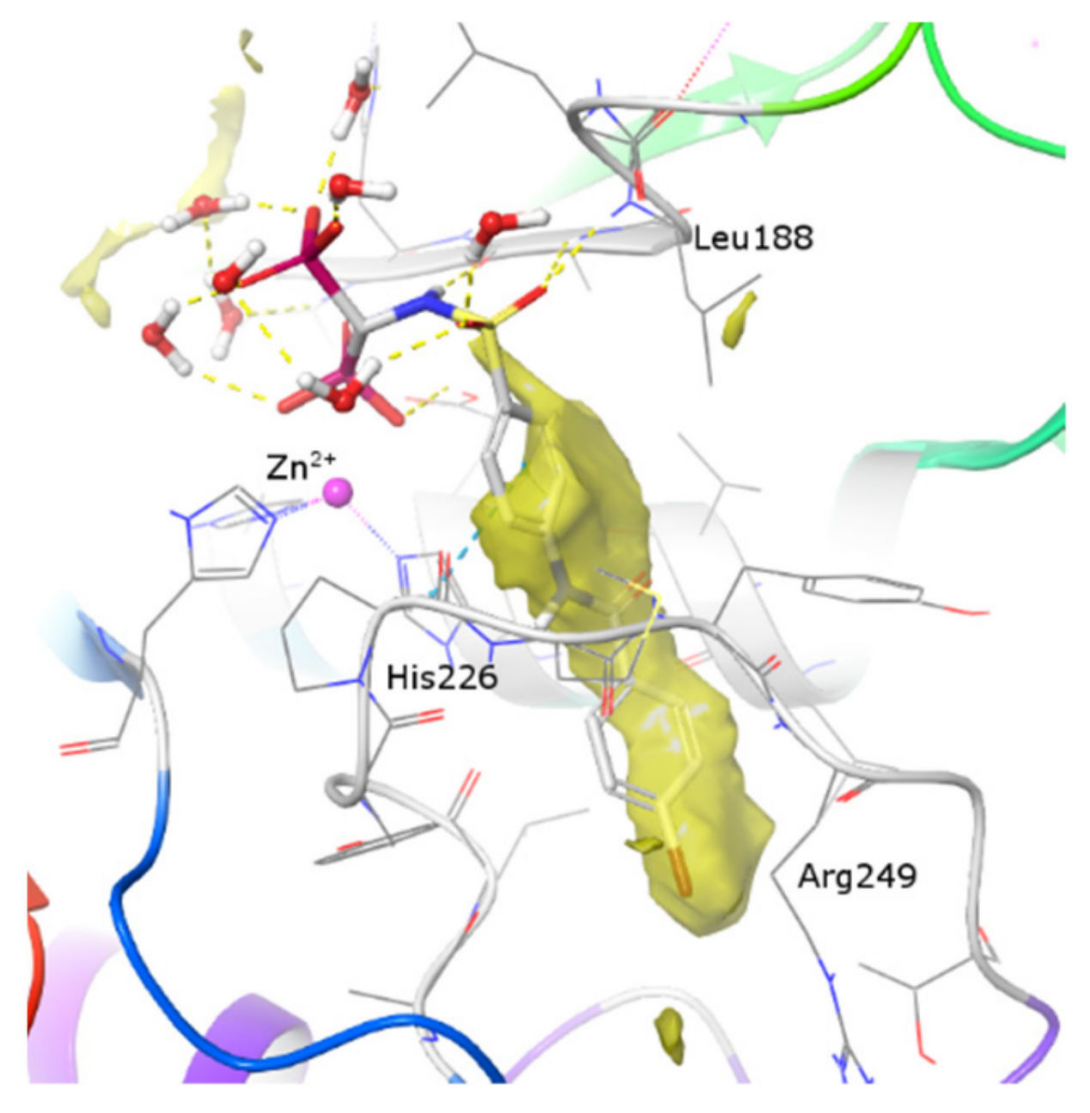
Superimposition of the docking pose of compound **9** (grey carbon atoms) to the hydrophobic molecular interaction fields (yellow transparent surface) calculated by SiteMap in the MMP-9 binding site (isovalue = −0.8, PDB ID: 4H3X).

The analysis of obtained docked poses shows that all ligands bind in the expected way, with the bisphosphonic function and the sulfonamide maintaining the crystallographic geometry of ML 115. Enzyme inhibition assays confirm the validity of the suggestion derived from the SiteMap analysis (Figure 3) to elongate the P1’ portion of new inhibitors in order to improve MMP-9 inhibition, since, indeed, newly synthesized compounds better occupy the hydrophobic S1’pocket. Compound **9**, in particular, almost perfectly fits in the hydrophobic field calculated by SiteMap for MMP-9, with the bromine substituent interacting with the Arg249 (Figure 5 and Figure S4). These positive interactions and the high complementarity with the hydrophobic field of the S1’ site can contribute to explaining the improved activity of this ligand with respect to ML 115 [36]. Therefore, we moved from an inactive hit (ML 115) to a nanomolar ligand (**9**) towards the target isoform MMP-9 by a more effective adaptation of the ligand to the large hydrophobic field in the enzyme S1’ site.

## 3. Materials and Methods 

### 3.1. MMP Inhibition Assays 

Catalytic domains of MMP-2, -8, -9, and -13 were obtained from from Enzo Life Sciences (Euroclone S.p.A., Pero, MI, Italy). Ninety-six-well white microtiter NBS plates (Corning, obtained from Merck Life Science S.r.l Milan, Italy) were used to carry out the assays (in triplicate). The assay measurements were performed by preparing dilutions to six different concentrations (1 nM–100 µM) of each inhibitor in a fluorometric assay buffer (50 mM Tris·HCl, pH 7.5, 200-mM NaCl, 1-mM CaCl_2_, 1-µM ZnCl_2_, 0.05% Brij-35, and 1% DMSO). Incubation of the enzyme and inhibitor solutions occurred for 15 min at room temperature; fluorogenic substrate solution (OmniMMP^®^ = Mca-Pro-Leu-Gly-Leu-Dpa-Ala-Arg-NH_2_, Enzo Life Sciences, 2.5-µM final concentration, or OmniMMP^®^RED = TQ3-GABA-Pro-Cha-Abu-Smc-His-Ala-Dab (6’-TAMRA)-Ala-Lys-NH_2_, Enzo Life Sciences (Euroclone S.p.A., Pero, MI, Italy), 1-µM final concentration) was subsequently added. The assay was incubated for 2–4 h at 37 °C, after which, a Perkin-Elmer Victor V3 plate reader (PerkinElmer, Waltham, MA, USA) was used to measure fluorescence (λex = 340 nm, λem = 405 nm or λex = 545 nm, λem = 572 nm) Included in the assay were control wells lacking any inhibitor. MMP activity was thus determined and expressed in relative fluorescence units (RFU). Percent inhibition was calculated from control wells. IC_50_ values were determined using GraphPad Prism 5.0 (GraphPad Software, La Jolla, CA, USA) and are shown as mean ± SEM of at least two independent measurements in triplicate.

### 3.2. Chemical Methods

Chemical reagents were commercially obtained from Merck Life Science S.r.l Milano, Italy and were utilized without purification. Monitoring of the reactions occurred via thin-layer chromatography (TLC; silica gel, UV_254_) with UV light (short-wave ultraviolet 254 nm and long-wave ultraviolet 365 nm). Inert atmosphere of N_2_ or Ar was used to carry out all reactions that required an anhydrous environment. Column chromatography was conducted using Fluka silica gel 60 Å (63–200 µm) or silica gel Si 60 (40–63 µm) (Merck Life Science S.r.l Milano, Italy). Mass spectrometry was conducted on a HP MS 6890-5973 MDS spectrometer, electron impact 70 eV, equipped with a HP ChemStation or with an Agilent 6530 Series Accurate-Mass Quadrupole Time-of-FLIFHT (Q-TOF) LC/MS (Agilent, Santa Clara CA, USA). A Bruker micro-TOF QII mass spectrometer (Bruker, Billerica, MA, USA) equipped with an electrospray ion source (ESI) was used to carry out high-resolution mass spectroscopy (HRMS) analyses. ^1^H NMR spectra were obtained deuterated solvents on a Varian Mercury 300 (Varian Inc., Palo Alto, CA, USA) or an Agilent VNMRS500 spectrometer (Agilent, Santa Clara CA, USA). Chemical shifts (δ) are expressed as parts per million (ppm) and coupling constants (J) in Hertz (Hz). Melting points were measured on a Gallenkamp electrothermal apparatus (Fisons Erba Science Ltd. Guildford, UK) in open capillaries and are uncorrected. 

#### 3.2.1. General Procedure for the Preparation of Sulfonamide Intermediates

A suspension of the appropriate sulfonyl chloride (4.41 mmol) and NH_4_OH (110.25 mmol) was stirred for 15 min at 0 °C and at room temperature for 3–24 h. Subsequently, the mixture was partitioned between EtOAc and NaHCO_3_, and the layers were separated. The organic phase was washed with brine, dried over anhydrous Na_2_SO_4_, filtered, and the filtrate was evaporated in vacuo to give a crude product that was used for the next step without any purification. 

**3-Nitrobenzenesulfonamide 14**: Yellow solid, 50% yield. ^1^H NMR (500 MHz, [D6]DMSO): δ = 7.65 (bs, 2H, NH_2_), 7.88 (t, J = 7.83, 1H, aromatic), 8.21–8.23 (m, 1H, aromatic), 8.43–8.45(m, 1H, aromatic), 8.57–8.58 (m, 1H, aromatic). GC-MS: *m*/*z* (%): 202(97), 186(33), 156(42), 138(59), 122(55), 108(25), 92(57), 75(100).

**4-Nitrobenzenesulfonamide 15**: Yellow solid, 50% yield; ^1^H NMR (400 MHz, [D6]DMSO): δ = 8.42–8.40 (m, 2H, aromatics), 8.07–8.05 (m, 2H, aromatics), 7.73 (bs, 2H, NH_2_). GC-MS: *m*/*z* (%): 202(100), 186(44), 156(20), 138(83), 122(60), 108(25), 92(35), 75(95).

**Naphthalene-1-sulfonamide 23**: White solid, 93% yield. ^1^H NMR (300 MHz, [D6]DMSO): δ = 7.59–7.68 (m, 3H, aromatics), 7.63 (bs, 2H, NH_2_), 8.06–8.16 (m, 3H, aromatics), 8.59–8.61 (m, 1H, aromatic). GC-MS: *m*/*z* (%): 207 (80), 143 (68), 127 (100), 115 (33).

**Naphthalene-2-sulfonamide 24**: White solid, 98% yield. ^1^H NMR (300 MHz, [D6]DMSO): δ = 7.49 (bs, 2H, NH_2_), 7.60–7.70 (m, 2H, aromatics), 7.85–7.87 (m, 1H, aromatic), 7.99–8.03 (m, 1H, aromatic), 8.09–8.14 (m, 2H, aromatics), 8.40–8.41 (m, 1H, aromatic). GC-MS: *m*/*z* (%): 207 (35), 143 (40), 127 (100), 115 (20), 77 (10).

#### 3.2.2. General Procedure for the Preparation of Tetraethyl Bisphosphonates 16, 17, 25, and 26

Triethyl orthoformate, diethyl phosphite, and the opportune sulfonamide were mixed in a 1.2:3:1 stoichiometric ratio. The reaction was heated at 160 °C until the evolution of EtOH was complete; then, EtOAc was used to dissolve the residue. Distillation of the solvent afforded a crude yellow oil, which was purified by column chromatography on silica gel (eluent EtOAc). The titled compounds were obtained as white solids in 35–50% yield.

**Tetraethyl ((3-nitro-phenylsulfonylamino)methyl)-1,1-bisphosphonate 16**: Yellow solid, 35% yield. ^1^H NMR (500 MHz, CDCl_3_): δ = 1.26–1.31 (m, 12H, CH_3_), 4.05–4.20 (m, 8H, CH_2_), 4.26 (td, J_HP_ = 22.00, J_HH_ = 9.78, 1H, CH), 5.80 (br, 1H, NH), 7.72 (t, J = 7.83, 1H, aromatic), 8.23–8.26 (m, 1H, aromatic), 8.43–8.431 (m, 1H, aromatic), 8.75 (t, J = 1.71, 1H, aromatic). MS(ESI)**:** *m*/*z*: 511 [M + Na]^+^, 489 [M + H]^+^; MS^2^ (489): *m*/*z* (%): 377 (35), 298 (64), 295 (64), 204 (26), 110 (100).

**Tetraethyl ((4-nitro-phenylsulfonylamino)methyl)-1,1-bisphosphonate 17**: Yellow solid, 47% yield; mp: 122–125 °C. ^1^H NMR (500 MHz, CDCl_3_): δ = 1.23–1.31 (m, 12 H, CH_3_), 4.11–4.21 (m, 8H, CH_2_), 4.25 (td, J_HP_ = 22.10, J_HH_ = 9.60, 1H, PCHP), 6.08 (br, 1H, NH), 8.08–8.12 (m, 2H, aromatics), 8.32–8.35 (m, 2H, aromatics); MS (ESI): *m*/*z*: 511 (M + Na]^+^; MS^2^: *m*/*z* (%): 373 (100).

**Tetraethyl ((1-naphthylsulfonylamino)methyl)-1,1-bisphosphonate 25**: White solid, 35% yield. ^1^H NMR(300 MHz, CDCl_3_): δ = 1.07–1.18 (m, 12H, CH_3_ ), 3.81–4.00 (m, 8H, CH_2_), 4.15 (td, J_HP_ = 22.00, J = 9.57, 1H, PCHP), 5.58 (br, 1H, NH), 7.54 (t, J = 7.84, 1H, aromatic), 7.58–7.63 (m, 1H, aromatic), 7.67–7.73 (m, 1H, aromatic), 7.92–7.96 (m, 1H, aromatic), 8.07 (d, J = 8.25, 1H, aromatic), 8.25 (dd, J_1_ = 7.43, J_2_ = 1.10, 1H, aromatic), 8.56 (d, J = 8.53, 1H, aromatic). MS (ESI): *m*/*z*: 516 [M + Na]^+^; MS^2^: *m*/*z* (%): 488 (39), 378 (100). 492[M − H]^−^; MS^2^: *m*/*z* (%): 191 (100). 

**Tetraethyl ((2-naphthylsulfonylamino)methyl)-1,1-bisphosphonate 26**: White solid, 50% yield. ^1^H NMR(300 MHz, CDCl_3_): δ = 1.12–1.23 (m, 12H, CH_3_), 3.90–4.12 (m, 8H, CH_2_), 4.23 (td, J_HP_ = 22.00, J_HH_ = 9.2, 1H, PCHP), 5.29 (br, 1H, NH), 7.57–7.67 (m, 2H, aromatics), 7.85–7.97 (m, 4H, aromatics), 8.43–8.44 (m, 1H, aromatic). MS (ESI): *m*/*z*: 516 [M + Na]^+^; MS^2^: *m*/*z* (%): 488 (28),378 (100). 492[M − H]^−^; MS^2^: *m*/*z* (%): 191 (100).

#### 3.2.3. General Procedure for Preparation of Amino Derivates 19 and 20 

To a solution of the nitro compound (0.532 mmol) in 8.5 mL EtOH, 10% Pd/C (0.20 mmol) was added. The hydrogenation of the mixture was carried out at room temperature at a pressure of 3 bar for 12–24 h. The reaction mixture was filtered through a celite pad, and the resulting solution was evaporated in vacuo, giving an oil. The crude was purified by chromatography on silica gel (eluent: CHCl_3_/MeOH 9.5:0.5 *v*/*v*), affording the desired amino derivate. 

**Tetraethyl ((3-amino-phenylsulfonylamino))methyl-1,1-bisphosphonate 18:** White solid, 75% yield. ^1^H NMR (300 MHz, CDCl_3_): δ = 1.11–1.43 (m, 12H, CH_3_), 3.99–4.07 (m, 1H, PCHP), 4.11–4.19 (m, 8H, CH_2_), 5.64 (br, 1H, NH), 6.85–6.87 (m, 1H, aromatic), 7.23–7.25 (m, 3H, aromatics). MS (ESI): *m*/*z*: 481[M + Na]^+^. 459[M + H]^+^; MS^2^(459): *m*/*z* (%): 183(100), 138(62).

**Tetraethyl ((4-amino-phenylsulfonylamino)methyl)-1,1-bisphosphonate 19:** White solid, 77% yield. ^1^H NMR (500 MHz, CDCl_3_): δ = 1.26–1.31 (m, 12H, CH_3_), 4.04–4.12 (m, 1H, PCHP), 4.13–4.20 (m, 8H, CH_2_), 5.05 (br, 1H, NH), 6.65 (dl, J = 8.81, 2H, aromatics), 7.65 (dl, J = 8.81, 2H, aromatics). MS (ESI): *m*/*z*: 481[M + Na]^+^, 459[M + H]^+^; MS^2^(469): *m*/*z* (%): 156(100), 138(28), 108(50).

#### 3.2.4. General Procedure for the Preparation of N-acylate Derivates 

The opportune acyl chloride (1.1–2 mmol) and triethylamine (2 mmol) were added to the solution of amino derivate (**18** and **19)** (1 mmol) in anhydrous THF. Stirring of the mixture occurred at room temperature under inert atmosphere (N_2_ or Ar) for 3–12 h. After a given time, the mixture was dried under vacuum and dissolved in EtOAc; the resulting solution was washed with NaHCO_3_ and then with HCl 1N, NH_4_Cl ss, and brine. The organic phase was finally dried over anhydrous Na_2_SO_4_, filtered, and the filtrate was evaporated in vacuo. The residue was purified by chromatography on silica gel (eluent: CHCl_3_/MeOH 98: 2 *v*/*v* or AcOEt /MeOH 9: 1 *v*/*v*) or crystallized with AcOEt to give the desired product. 

**Tetraethyl((3-benzamidophenylsulfonylamino)methyl)-1,1-bisphosphonate 6a:**

White solid, 64% yield, (AcOEt). ^1^H NMR (500MHz, [D_6_] DMSO): δ = 1.11–1.88 (m, 12H, CH_3_), 3.87–4.03 (m, 8H, CH_2_), 4.11 (t, J_HP_ = 23.00, 1H, PCHP), 7.50–7.61 (m, 5H, aromatics), 7.95–7.97 (m, 3H, aromatics), 8.30 (s, 1H, aromatic), 8.80 (br, 1H, NH), 10.50 (s, 1H, NH). MS (ESI): *m*/*z*: 561[M − H]^−^; MS^2^: *m*/*z* (%): 288(54), 260(37), 134 (100), 106 (34). 563 [M + H]^+^.

**Tetraethyl((4-benzamidophenylsulfonylamino)methyl)-1,1-bisphosphonate 7a:**

White solid, 65% yield (chromatography, eluent: CHCl_3_/MeOH 98: 2 *v*/*v*). ^1^H NMR (500 MHz, [D_6_] DMSO): δ = 1.13–1.17 (m, 12H, CH_3_), 3.89–4.05 (m, 8H, CH_2_), 4.14 (t, J_HP_ = 23.00, 1H, PCHP), 7.52–7.62 (m, 3H, aromatics), 7.78 (d, J = 8.81, 2H, aromatics), 7.90–7.96 (m, 4H, aromatics), 8.63 (br, 1H, NH), 10.53 (s, 1H, NH). MS (ESI): *m*/*z*: 585[M + Na]^+^, 563[M + H]^+^; MS^2^(563): *m*/*z* (%): 351(22), 282(10), 261(20), 260(100), 196(50), 166(63), 138(74), 110(42).

**Tetraethyl((4-(4-methylbenzamido)phenylsulfonylamino)methyl)-1,1-bisphosphonate 8a:**

White solid, 79% yield (AcOEt). ^1^H NMR(500 MHz, CDCl_3_): δ = 1.25–1.34 (m, 12H, CH_3_), 2.43 (s, 3H, CH_3_), 4.06–4.20 (m, 8H, CH_2_), 4.23 (td, J_HP_ = 22.00, J = 9.79, 1H, PCHP), 5.31 (br, 1H, NH), 7.30 (dl, J = 7.83, 2H, aromatics), 7.78–7.88 (m, 6H, aromatics), 8.15 (bs, 1H, NH). MS (ESI): *m*/*z*: 599 [M + Na]^+^, 577[M + H]^+^; MS^2^(577): *m*/*z* (%): 393(18), 365(23), 291(21), 275(26), 274(100), 166(87), 138(74), 138(58), 119 (96), 110 (36). 575[M − H]^−^; MS^2^: *m*/*z* (%): 437(25), 302(16), 210(32), 134(100). 

**Tetraethyl((4-(4-bromobenzamido)phenylsulfonylamino)methyl)-1,1-bisphosphonate 9a:**

White solid, 55% yield (AcOEt). ^1^H NMR(500 MHz, CDCl_3_): δ = 1.14–1.18 (m, 12H, CH_3_), 3.94- 4.04 (m, 8H, CH_2_), 4.14 (t, J_HP_ = 23.00, 1H, PCHP), 7.74 (d, J = 8.32, 2H, aromatics), 7.79 (d, J = 8.81, 2H, aromatics), 7.80–7.92 (m, 4H, aromatics), 8.55 (br, 1H, NH), 10.54 (s, 1H, NH). MS (ESI): *m*/*z*: 643[M + 2 + H]^+^, 641[M + H]^+^; MS^2^(641): *m*/*z* (%): 458(18), 431(18), 340(59), 338(53), 276 (11), 274(15), 185(24), 183(32), 166(100), 138(82), 110(39). 

**Tetraethyl((4-(3-bromobenzamido)phenylsulfonylamino)methyl)-1,1-bisphosphonate 10a:**

White solid, 73% yield (AcOEt). ^1^H NMR(500 MHz, [D_6_] DMSO): δ = 1.13–1.96 (m, 12H, CH_3_), 3.97–4.05 (m, 8H, CH_2_), 4.14 (t, J_HP_ = 23.00, 1H, PCHP), 7.51 (t, J = 8.08, 1H, aromatic), 7.80–7.82 (m, 3H, aromatics), 7.89–7.96 (m, 3H, aromatics), 8.14 (t, J = 1.96, 1H, aromatic), 8.63 (br, 1H, NH), 10.62 (s, 1H, NH). MS (ESI): *m*/*z*: 643[M+2+H]^+^, 641 [M + H]^+^; MS^2^: *m*/*z* (%): 459(12), 430(12), 340(70), 338(43), 276(20), 274(18), 185(18), 183 (42), 166(88), 138(100). 

**Tetraethyl((4-(4-nitrobenzamido)phenylsulfonylamino)methyl)-1,1-bisphosphonate 11a:**

White solid, 57% yield (AcOEt). ^1^H NMR(500 MHz, [D_6_] DMSO): δ = 1.15–1.17 (m, 12H, CH_3_), 3.90–4.05 (m, 8H, CH_2_), 4.14 (t, J_HP_ = 23.00, 1H, PCHP), 7.81 (d, J = 8.56, 2H, aromatics), 7.92 (d, J = 8.56, 2H, aromatics), 8.17–8.20 (m, 2H, aromatics), 8.36–8.39 (m, 2H, aromatics), 8.66 (br, 1H, NH), 10.85 (s, 1H, NH). MS (ESI): *m*/*z*: 630[M + Na]^+^, 608 [M + H]^+^; MS^2^(630): *m*/*z* (%): 492(100). 606 [M − H]^−^; MS^2^: *m*/*z* (%): 468(86), 333(42), 305(45), 240 (100), 134(61). 

**Tetraethyl((4-(1-naphthamido)phenylsulfonylamino)methyl)-1,1-bisphosphonate 12a:**

White solid, 59% yield (AcOEt). ^1^H NMR(500 MHz, CDCl_3_): δ = 1.16–1.43 (m, 12H, CH_3_), 4.04–4.15 (m, 8H, CH_2_), 4.23 (t, J_HP_ = 22.02, J_HH_ = 9.79, 1H, PCHP), 5.29 (br, 1H, NH), 7.52 (t, J = 7.59, 1H, aromatic), 7.55–7.62 (m, 2H, aromatics), 7.74 (d, J = 6.85, 1H, aromatics), 7.81–7.88 (m, 2H, aromatics), 7.88–7.97 (m, 3H, aromatics), 7.99 (d, J = 8.32, 1H, aromatics), 8.10 (s, 1H, NH), 8.30 (d, J = 7.83, 1H, aromatic). MS (ESI): *m*/*z*: 635[M + Na]^+^, 613 [M + H]^+^; MS^2^(613): *m*/*z* (%): 310(47), 166(55), 155(100), 138(45), 110(22). 611[M − H]^−^; MS^2^: *m*/*z* (%): 473(45), 388(67), 310(42), 246 (33), 245(39), 134 (100), 106(29). 

**Tetraethyl((4-(2-naphthamido)phenylsulfonylamino)methyl)-1,1-bisphosphonate 13a:**

White solid, 49% yield (chromatography, eluent: AcOEt/MeOH 9:1 *v*/*v*). ^1^H NMR (500 MHz, [D_6_] DMSO): δ = 1.04–1.29 (m, 12H, CH_3_), 3.89–4.09 (m, 8H, CH_2_), 4.16 (t, J_HP_ = 22.75, 1H, PCHP), 7.60–7.67 (m, 2H, aromatics), 7.82 (d, J = 8.81, 2H, aromatics), 7.96–8.10 (m, 6H, aromatics), 8.60 (s, 1H, aromatic), 8.63 (br, 1H, NH), 10.72 (s, 1H, NH). MS (ESI): *m*/*z*: 635[M + Na]^+^, 613 [M + H]^+^; MS^2^(613): *m*/*z* (%): 310(76), 166(61), 134(54), 110(18). 611[M − H]^−^; MS^2^: *m*/*z* (%): 473(43), 388(70), 310(30), 246 (25), 245(33), 134 (100), 106(29). 

**Tetraethyl((4-(3 phenylureido)phenylsulfonylamino)methyl)-1,1-bisphosphonate 4a:**

A solution of phenyl isothiocyanate (1.2 mmol) in anhydrous toluene (2 mL) was added to the suspension of **19** (1 mmol) in anhydrous toluene (2 mL), and the mixture was heated to reflux for 2 h. The eluent was evaporated in vacuo, and the residue was purified by chromatography on silica gel (eluent AcOEt/MeOH 9: 1 *v*/*v*) to obtain the desired product. 

White solid, 71% yield. ^1^H NMR (500 MHz, CDCl_3_): δ = 1.17–1.81 (m, 12H, CH_3_), 4.05–4.16 (m, 8H, CH_2_), 4.31 (td, J_HP_ = 22.26, J_HH_ = 9.30, 1H, PCHP), 5.75 (br, 1H, NH), 7.03 (t, J = 7.34, 1H, aromatics), 7.26–7.28 (m, 2H, aromatics), 7.42 (d, J = 7.34, 2H, aromatics), 7.58 (d, J = 6.85, 2H, aromatics), 7.72 (d, J = 7.34, 2H, aromatics), 8.09 (bs, 1H, NH), 8.39 (bs, 1H, NH). MS(ESI): *m*/*z*: 600[M + Na]^+^, 578[M + H]^+^; MS^2^(578): *m*/*z* (%): 366(13), 293(22), 275 (100), 211(36), 166 (84), 156(24), 138 (49), 108(40).

**Tetraethyl((4-(1,3-dioxoisoindolin-2-yl)phenylsulfonylamino)methyl)-1,1-bisphosphonate 5a:**

A mixture of **19** (1 mmol) and phthalic anhydride (1.07 mmol) in 6 ml of glacial acetic acid was refluxed for 4 h. The resulting solution was dried under vacuum. EtOAc was subsequently used to dilute the residue; then, 6-M NaOH was added until pH = 6. After separation of the layers, the organic phase was washed with brine, dried over anhydrous Na_2_SO_4_, filtered, and the filtrate was dried in vacuo. Column chromatography on silica gel (eluent: CHCl_3_/MeOH 9: 1 *v*/*v*) was used to purify the residue, affording the desired product. 

White solid, 47% yield. ^1^H NMR (500 MHz, [D_6_]DMSO): δ = 1.15–1.17 (m, 12H, CH_3_), 3.87–4.05 (m, 8H, CH_2_), 4.16 (t, J = 23.00, 1H, PCHP), 7.61(d, J = 8.32, 2H, aromatics), 7.90–7.98 (m, 6H, aromatics), 8.89 (br, 1H, NH). MS (ESI): *m*/*z*: 611[M + Na]^+^; MS^2^: *m*/*z* (%): 473 (100), 445(11), 324(10), 270(23), 160 (12). 587[M − H]^−^; MS^2^: *m*/*z* %: 287(12), 286(60), 222 (31), 137(100), 108(30). 

#### 3.2.5. General Procedure for the Preparation of 1,1-bisphosphonic Acids

**Method A**: A solution of the appropriate tetraethyl bisphosphonate (1 mmol) in 4-mL 2N HCl solution was refluxed for 12–24 h. The aqueous phase was removed under reduced pressure, and the crude bisphosphonic acids were then triturated with the opportune solvent and filtered to afford the final compounds as white solids in 20–96% yield.

**Method B**: Anhydrous trimethylsilylbromide (17–32 mmol) was carefully added to a solution of the corresponding tetraethyl bisphosphonate (1 mmol) in anhydrous acetonitrile (6 mL) at 0 °C under argon, and the resulting mixture was stirred at room temperature for 24–48 h. Two milliliters of MeOH were added, and the mixture was stirred for 5 min; the solvent was distilled off, and the crude bisphosphonic acids were triturated with the opportune solvent and filtered to afford the desired compounds as white solids in a 33–99% yield. 

**(4-aminophenylsulfonylamino)methyl-1,1-bisphosphonic acid (1). Method B:**

White solid, mp: 243°C (dec)(Acetone/ H_2_O). ^1^H NMR (500 MHz, [D6]DMSO): δ = 3.78 (td, J_HP_= 21.53, J = 9.30, 1H, PCHP), 5.38–6.42 (br, 6H, OH, NH_2_), 6.50 (d, J = 8.32, 2H, aromatics), 6.81(d, J = 9.30, 1H, NH), 7.45 (d, J = 8.32, 2H, aromatics). ^31^P NMR (500 MHz, [D_6_] DMSO): δ = 15.03 (d, J_PH_ = 22.84). MS (ESI): *m*/*z*: 345 [M − H]^−^; MS^2^: *m*/*z* (%): 280 (100). HRMS [M − H]^−^: calculated 344.9717; found 344.9709.

**(2-naphthylsulfonylamino)methyl-1,1-bisphosphonic acid (2). Method B:**

White solid, 43% yield. (IPA) mp: 107–110 °C; ^1^H NMR (300 MHz, [D6]DMSO): δ = 3.88 (td, J_HP_= 21.86, J_HH_ = 9.63, 1H, PCHP), 6.2–7.00 (br, 4H, OH), 7.60–7.66 (m, 2H, aromatics), 7.90–8.07 (m, 5H, 4H aromatics, NH), 8.40 (s, 1H, aromatic). ^31^P NMR (300 MHz, [D_6_] DMSO): δ = 14.96 (d, J_PH_ = 21.37). MS (ESI): *m*/*z*: 380 [M − H]; MS^2^: *m*/*z* (%): 362 (100), 298(19). 

**(1-naphthylsulfonylamino)methyl-1,1-bisphosphonic acid (3). Method A:**

White solid, 20% yield; mp: 183 °C (dec) (MeOH/Et_2_O 1:1 *v*/*v*). ^1^H NMR (300MHz, [D6]DMSO): δ = 3.71 (t, J_HP_= 19.52, 1H, PCHP), 6.10–6.50 and 6.99–7.38 (br, 5H, 4 OH and NH), 7.51 (t, J = 7.70, 1H, aromatic), 7.55–7.65 (m, 2H, aromatics), 7.98–8.02 (m, 1H, aromatic), 8.08 (d, J = 8.25, 1H, aromatic), 8.36 (d, J = 6.88, 1H, aromatic), 8.64 (d, J = 7.98, 1H, aromatic). ^31^P NMR (300 MHz [D_6_] DMSO): δ = 14.14 (d, J_PH_ = 18.34). MS (ESI): *m*/*z*: 380 [M − H]^−^; MS^2^: *m*/*z* (%): 362 (100), 298 (21). 

**(4-(3-phenylureido)phenylsulfonylamino)methyl-1,1-bisphosphonic acid (4). Method B:**

White solid, 33% yield; mp: > 243 °C (dec). ^1^H NMR (500 MHz, [D6]DMSO): δ = 3.82 (td, J_HP_ = 22.00, J = 9.30, 1H, PCHP), 5.65–6.80 (br, 5H, OH and NH), 6.97 (t, J = 7.34, 1H, aromatic), 7.27 (t, J = 7.34, 2H, aromatics), 7.42–7.54 (m, 4H, aromatics), 7.72 (d, J = 7.82, 2H, aromatics), 8.76 (s, 1H, NH), 9.01(s, 1H, NH). ^31^P NMR (500 MHz, [D6]DMSO): δ = 15.04 (d, J_PH_ = 21.25). MS (ESI): *m*/*z*: 464[M − H]^−^; MS^2^: *m*/*z* (%): 382(56), 275(27), 263(95), 106(71), 79(100). HRMS [M − H]^−^: calculated 464.0088; found 464.0081.

**(4-(1,3-dioxoisoindolin-2-yl)phenylsulfonylamino)methyl-1,1-bisphosphonic acid (5). Method B:**

White solid, 47% yield; mp: > 250 °C (MeOH). ^1^H NMR (500 MHz, [D6] DMSO): δ = 3.87–3.97 (td, J_HP_ = 21.53, J = 9.46, 1H, PCHP), 4.25–5.40 (br, 5H, OH and NH), 7.56 (d, J = 8.30, 2H, aromatics), 7.89–7.93 (m, 3H, aromatics), 7.96–8.00 (m, 3H, aromatics). ^31^P NMR (500 MHz, [D6]DMSO): δ = 14.90 (d, J_PH_ = 21.36). MS (ESI): *m*/*z*: 475[M − H]^−^; MS^2^: *m*/*z* (%): 393(100), 286(65), 106(100). HRMS [M − H]^−^: calculated 474.9772; found 474.9768.

**(4-benzamidophenylsulfonylamino)methyl-1,1-bisphosphonic acid (6). Method B:**

White solid, 99% yield; mp: 243–245 °C (Acetone). ^1^H NMR (500 MHz, [D6]DMSO): δ = 3.83 (td, 1H, J_HP_ = 21.53, J = 9.54, 1H, PCHP), 4.0–5.5 (br, 4H, OH), 7.53 (t, J = 7.34, 2H, aromatics), 7.58–7.60 (m, 2H, 1H aromatic, NH), 7.79 (d, J = 8.81, 2H, aromatics), 7.87 (d, J = 8.87, 2H, aromatics), 7.94 (d, J = 7.34, 2H, aromatics), 10.54 (s, 1H, NH). ^31^P NMR (500 MHz, [D6]DMSO): δ = 14.82 (s). MS (ESI): *m*/*z*: 449[M − H]^−^; MS^2^: *m*/*z* (%): 368(20), 367(100), 260(45), 106(76). HRMS [M − H]^−^: calculated 448.9979; found 448.9972.

**(3-benzamidophenylsulfonylamino)methyl-1,1-bisphosphonic acid (7). Method B:**

White solid, 53% yield; mp: > 250 °C (IPA). ^1^H NMR (300 MHz, [D6]DMSO): δ = 3.75–3.97 (m, 1H, PCHP), 5.65–6.57 (br, 4H, OH), 7.41–7.65 (m, 5H, aromatics), 7.67–7.80 (br, 1H, NH), 7.95–7.98 (m, 3H, aromatics), 8.25 (s, 1H, aromatic), 10.43 (s, 1H, NH). ^31^P NMR (500 MHz, [D6]DMSO): δ = 14.87 (d, J_PH_ = 19.82). MS (ESI): *m*/*z*: 449 [M − H]^−^; MS^2^: *m*/*z* (%): 367(94), 260(34), 106(100). HRMS [M − H]^−^: calculated 448.9979; found 448.9973.

**(4-(4-methylbenzamido)phenylsulfonylamino)methyl-1,1-bisphosphonic acid (8). Method B:**

White solid, 61% yield; mp: > 250 °C (MeOH). ^1^H NMR (500 MHz, [D6]DMSO): δ = 2.38 (s, 3H, CH_3_), 3.84 (td, J_HP_ = 21.75, J = 8.90, 1H, PCHP), 5.18–6.05 (br, 5H, OH, NH), 7.34 (d, J = 7.83, 2H, aromatics), 7.58 (d, J = 8.32, 1H, aromatic), 7.78–7.87 (m, 5H, aromatics), 10.40 (s, 1H, NH). ^31^P NMR (500 MHz, [D6]DMSO): δ = 14.02 (d, J_PH_ = 21.25). MS (ESI): *m*/*z*: 463 [M − H]^−^; MS^2^: *m*/*z* (%): 382(14), 381(62), 274(34), 106(60), 79(100). HRMS [M − H]^−^: calculated 463.0135; found 463.0130.

**(4-(4-bromobenzamido)phenylsulfonylamino)methyl-1,1-bisphosphonic acid (9) Method B:**

White solid, 80% yield; mp: > 250 °C (MeOH). ^1^H NMR (300 MHz, [D6]DMSO): δ = 3.74–3.92 (td, J_HP_ = 21.67, J = 9.66, 1H, PCHP), 7.46–7.92 (m, 12H, 4OH, 8 H aromatics), 7.67 (d, J = 9.66, 1H, NH), 10.56 (s, 1H, NH). ^31^P NMR (500 MHz, [D6]DMSO): δ =14.95 (d, J_PH_ = 21.36). MS(ESI): *m*/*z*: 529[M + 2 − H]^−^, 527[M − H]^−^. MS^2^: *m*/*z* (%): 447(100), 445(69), 340(24), 338(19). HRMS [M − H]^−^: calculated 526.9084; found 526.9077.

**(4-(3-bromobenzamido)phenylsulfonylamino)methyl-1,1-bisphosphonic acid (10) Method B:**

White solid, 68% yield; mp: >246 °C (IPA). ^1^H NMR (500 MHz, [D6]DMSO): δ = 3.83 (td, 1H, J_HP_ = 21.76, J= 9.29, 1H, PCHP), 6.20–7.07 (br, 4H, OH), 7.50 (t, J = 7.83, 1H, aromatic), 7.63 (d, J = 9.29, 1H, NH), 7.80–7.86 (m, 5H, aromatics), 7.94 (d, J = 7.83, 1H, aromatic), 8.12–8.13 (m, 1H, aromatic), 10.58 (s, 1H, NH). ^31^P NMR (500 MHz [D6]DMSO): δ = 14.92 (d, J_PH_ = 21.24). MS (ESI): *m*/*z*: 529[M + 2 − H]^−^, 527[M − H]^−^; MS^2^: *m*/*z* (%): 447(98), 445 (69), 340(23), 338(20), 106 (72), 79 (100). HRMS [M − H]^−^: calculated 526.9084; found 526.9076.

(**4-(4-nitrobenzamido)phenylsulfonylamino)methyl-1,1-bisphosphonic acid (11) Method B: **

White solid, 71% yield; mp: > 250 °C (MeOH). ^1^H NMR (500 MHz, [D6]DMSO): δ = 3.79–3.90 (td, J_HP_ = 22.00, J = 9.45, 1H, PCHP), 6.00–7.20 (br, 4H, OH), 7.69 (d, J = 9.45, 1H, NH), 7.81–7.88 (m, 4H, aromatics), 8.18 (d, J = 8.81, 2H, aromatics), 8.37 (d, J = 8.81, 2H, aromatics), 10.80 (s, 1H, NH). ^31^P NMR (500 MHz [D6]DMSO): δ = 14.96 (d, J_PH_ = 21.36). MS (ESI): *m*/*z*: 494[M − H]^−^MS^2^: *m*/*z* (%): 458 (8), 412(100), 305 (56), 106 (76). HRMS [M − H]^−^: calculated 493.9830; found 493.9824.

**(4-(1-naphthamido)phenylsulfonylamino)methyl-1,1-bisphosphonic acid (12) Method B:**

White solid, 44% yield; mp: 237–240 °C (IPA). ^1^H NMR (500 MHz, [D6]DMSO): δ = 3.86 (t, J_HP_ = 21.52, J = 9.27, 1H, PCHP), 4.25–6.22 (br, 4H, OH), 7.57–7.62 (m, 4H, 3H aromatics, NH), 7.76 (d, J = 6.85, 1H, aromatic), 7.82 (d, J = 8.81, 2H, aromatics), 7.88 (d, J = 8.81, 2H, aromatics), 8.00–8.03 (m, 1H, aromatic), 8.09 (d, J = 8.39, 1H, aromatic), 8.13–8.17 (m, 1H, aromatic), 10.85 (s, 1H, NH). ^31^P NMR (500 MHz, [D6]DMSO): δ = 13.91 (d, J_PH_ = 21.45). MS (ESI): *m*/*z*: 499 [M − H]^−^; MS^2^: *m*/*z* (%): 417 (94), 310(39), 106(66), 79(100). HRMS [M − H]^−^: calculated 499.0135; found 499.0130.

**(4-(2-naphthamido)phenylsulfonylamino)methyl-1,1-bisphosphonic acid (13) Method B:**

White solid, 68% yield; mp: 248–250 °C (IPA). ^1^H NMR (500 MHz, [D6]DMSO): δ = 3.85 (td, J_HP_ = 22.01, J = 9.78, 1H, PCHP), 5.15–5.95 (br, 4H, OH), 7.53–7.58 (br, 1H), 7.60–7.66 (m, 2H, aromatics), 7.83 (d, J = 9.30, 2H, aromatics), 7.92 (d, J = 8.81, 2H, aromatics), 7.99–8.09 (m, 4H, aromatics), 8.58 (s, 1H), 10.67 (s, 1H, NH). ^31^P NMR (500 MHz, [D6]DMSO): δ = 13.97 (d, J_PH_ = 21.25). MS (ESI): *m*/*z*: 499 [M − H]^−^; MS^2^: *m*/*z* (%): 417 (90), 310 (37), 106 (66), 79 (100). HRMS [M − H]^−^: calculated 499.0135; found 499.0127.

### 3.3. Computational Studies

All calculations were carried out using the Schrodinger Suite 2018-3 (Schrodinger, New York, NY, USA) [31]. Ligand structures were built in Maestro. A fully deprotonated form of the bisphosphonic function was generated. All ligands were minimized at a convergence gradient of 0.05 using the PRCG minimization algorithm. Receptor structures were retrieved from the Protein Data Bank. Complexes with PDB ID: 1QIB, 3DPF, 4H3X, and 2OZR, other than our structure 4KQZ, were selected respectively for MMP-2, MMP-8 (with larger S1’ site), MMP-9, and MMP-13. All structures were prepared for successive calculations using the Protein Preparation Wizard of Maestro that fix the bond order, the protonation state, and the water molecules. The catalytic glutamate was kept protonated to guarantee the H-bond interaction with one phosphonic group. Water molecules directly bound to ML115 in the X-ray complex with MMP-8 were maintained and minimized with the ligand in the receptor structure. These 9 water molecules were copied with the ligand ML115 in its X-ray position to the other MMPs under study. In each MMP, added water molecules and ligand were submitted to a constrained minimization to optimize their position and H-bonding. After minimization, the ligand was deleted, and Glide Grid Generation tool was applied to obtain the grid file necessary for subsequent docking calculations. Docking was performed using Glide (Schrodinger, New York, NY, USA) and exploiting the virtual screening workflow. Standard precision (SP), extra precision (XP), and MM-GBSA calculations were set up, imposing an extended conformational sampling for the ligands. Enrichment was used as a validation tool of the docking protocol. ROC (Receiver Operating Characteristic) values were calculated considering the active compounds with pIC50 > 6. Results are reported in Table S2. SiteMap calculations were carried out on the MMP-9 S1’ site using a restrictive definition of hydrophobicity and calculating a fine grid.

## 4. Conclusions

In conclusion, a series of novel bisphosphonic compounds was synthesized and tested on a panel of MMP isozymes with the aim of improving the activity profile of reference compound ML 115 towards MMP-9.

The benzensulfonylamide scaffold of ML 115 was derivatized with different substituents in order to achieve the same hydrophobic interaction shown by its diphenyl moiety. The best results were obtained when an amide spacer was introduced as a structural flexibility element, as seen with compound **6**.

Further developments led to the obtainment of compounds, such as **9** and **11**, with a bulky electron-withdrawing substituent in the 4’ position (bromine and nitro, respectively) with even lower IC_50_ towards MMP-9. Compound **9**, in particular, proved to be the best in the series, considering its high potency towards MMP-9 while maintaining a good activity for the other isoforms. On the other hand, better selectivity towards MMP-2 was reached for compounds **4** and **5**, which showed a proper activity profile, sparing MMP-8. These results show that the design of BMMPIs targeting MMP-2 and -9 is indeed possible and represents a promising avenue of research for the treatment of bone metastases, which could maximize therapeutic efficacy while limiting side effects.

## Figures and Tables

**Table 1:** Inhibition data for compounds **1–13** against matrix metalloproteinases (MMPs).

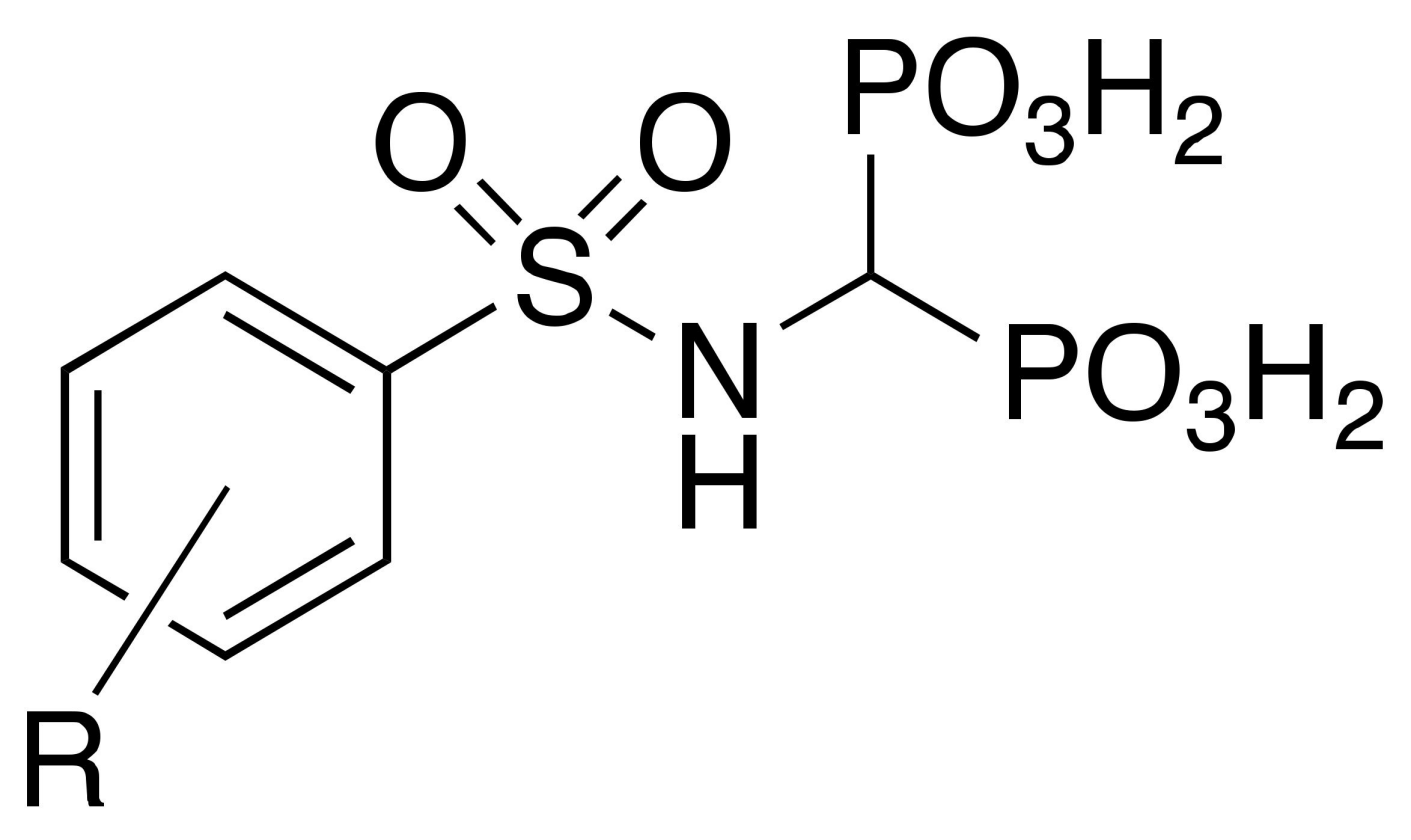		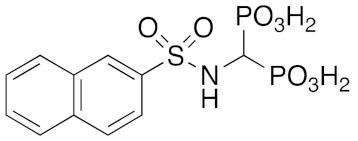		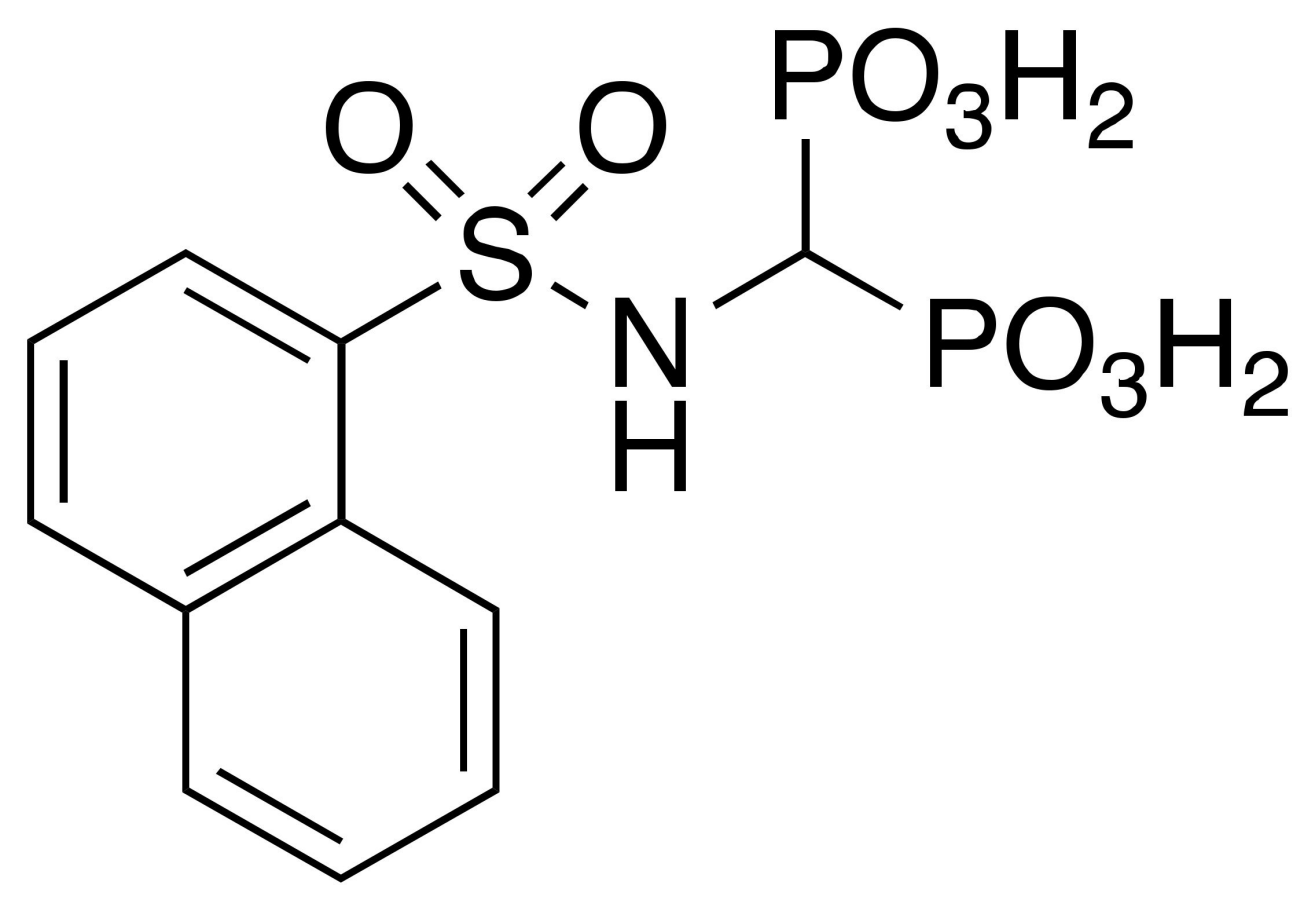	
**1,4–13**		**2**		**3**	
**Compd.**	**R**	**MMP-2**	**MMP-8**	**MMP-9**	**MMP-13**
1	NH_2_	19 ± 3	66 ± 8	>100	68.9 ± 10.17
ML 115	4-Ph	0.14 ± 0.04	0.40 ± 0.03	>100	0.74 ± 0.06
2	-	18.7 ± 0.8	26.8 ± 0.9	>100	9.6 ± 0.4
3	-	15.2 ± 0.5	31.3 ± 1.2	57 ± 2	14.6 ± 0.8
4	4-NHCONHPh	2.8 ± 0.6	48 ± 15	13.8 ± 0.6	15 ± 6
5	4-N-Phthalimido	5.3 ± 0.4	45 ± 5	15 ± 6	18.1 ± 1.5
6	4-NHCOPh	2.75 ± 0.35	1.88 ± 0.03	2.3 ± 0.1	2.0 ± 0.7
7	3-NHCOPh	9.0 ± 0.8	79.0 ± 8.0	50 ± 11	28 ± 4
8	4-NHCOPh-4’-CH_3_	0.958 ± 0.018	0.78 ± 0.18	0.67 ± 0.25	3.3 ± 0.7
9	4-NHCOPh-4’-Br	0.67 ± 0.16	0.66 ± 0.13	0.088 ± 0.012	0.14 ± 0.05
10	4-NHCOPh-3’-Br	4.2 ± 1.3	17 ± 4	>100	15.9 ± 1.9
11	4-NHCOPh-4’-NO_2_	1.7 ± 0.3	0.46 ± 0.11	0.43 ± 0.10	0.61 ± 0.16
12	4-NHCO-1-naphthyl	2.7 ± 1.0	25 ± 12	21.5 ± 0.7	12 ± 5
13	4-NHCO-2-naphthyl	3.9 ± 0.5	16.6 ± 1.9	76 ± 8	9.3 ± 0.5

Inhibitory activity was evaluated by fluorometric assay using commercially available catalytic domain of MMP-2, -8, -9, and -13. IC50 µM values are reported as the mean ± std. deviation of at least 2 independent experiments performed in triplicate.

## Supplementary Materials

The following are available online at https://www.mdpi.com/1424-8247/13/6/113/s1: Table S1: statistics of crystallographic data and refinement for crystals of MMP-8 in complex with ML115. Figure S1: 2F_o_-F_c_ electron density map calculated around ML115. Figure S2: Superposition of the previously obtained X-ray complex MMP-8: monophosphonate sulfonamide with the MMP-8:ML 115. Table S2: enrichment values obtained ranking the docked compounds on the basis of calculated MM-GBSA DG bind. Figure S3: ROC curves obtained for inhibitor docked poses into MMP-2, MMP-8 (4KQZ), MMP-8 (3DPF), MMP-9 and MMP-13. 3. Figure S4: 2D ligand interaction diagram representing the contacts between ligand 9 docked in the MMP-9 binding site.
