# Supplementary material for: Bone-Seeking Matrix Metalloproteinase Inhibitors for the Treatment of Skeletal Malignancy
Source: Pharmaceuticals (Basel). 2020 Jun 1;13(6):113. doi: 10.3390/ph13060113 (PMC7344628; doi:10.3390/ph13060113)
Supplement: Supplementary file 1 [file pharmaceuticals-13-00113-s001.pdf]

pharmaceuticals

MDPI

Article

Supplementary Materials of Bone-Seeking Matrix Metalloproteinase Inhibitors for the Treatment of Skeletal Malignancy.

Antonio Laghezza [1], Luca Piemontese [1], Leonardo Brunetti [1], Alessia Caradonna [1], Mariangela Agamennone [2], Antonella Di Pizio [3], Giorgio Pochetti [4], Roberta Montanari [4], Davide Capelli [4], Marilena Tauro [5], Fulvio Loiodice [1,*] and Paolo Tortorella [1,*]

1 Department of Pharmacy and Pharmaceutical Sciences, University of Bari "A. Moro", via E. Orabona 4, 70125 Bari, Italy; antonio.laghezza@uniba.it (A.L.); luca.piemontese@uniba.it (L.P.); l.brunetti2@studenti.uniba.it (L.B.); a.caradonna@hotmail.it (A.C.)
2 Department of Pharmacy, University "G. d'Annunzio" of Chieti-Pescara, Via Dei Vestini, 31, 66100 Chieti, Italy; magamennone@unich.it
3 Leibniz-Institute for Food Systems Biology at the Technical University of Munich, Lise-Meitner-Str. 34, 85354 Freising, Germany; antonelladipizio@gmail.com
4 Istituto di Cristallografia, Consiglio Nazionale delle Ricerche, Montelibretti, Monterotondo Stazione, Roma 00015 Italy; giorgio.pochetti@ic.cnr.it (G.P.); roberta.montanari@ic.cnr.it (R.M.); davide.capelli@ic.cnr.it (D.C.)
5 Tumor Biology Department, H. Lee Moffitt Cancer Center and Research Institute, Tampa, 33612, USA; marilena.tauro@moffitt.org
* Correspondence: fulvio.loiodice@uniba.it (F.L.); paolo.tortorella@uniba.it (P.T.)

Expression and Purification of the Protein

The truncated form M80-G242 of the catalytic domain of MMP-8 was expressed in *E. coli* BL21 (DE3). The culture was induced with 0.5 mM IPTG when OD_{600} = 0.5 - 0.6 and further incubated at 310 K for 3 hours. Inclusion bodies isolated and purified from harvested *E. coli* cells were resuspended in Tris 20 mM, pH 8.5, Urea 6 M, β-mercaptoethanol 100 mM and incubated overnight at room temperature under shaking to extract the solubilized collagenase. This extract was centrifuged for 30′ at 40000 rpm, and the supernatant was loaded onto a Mono Q-Sepharose column (GE Healthcare) previously equilibrated with the denaturating buffer.

Elution of the collagenase was carried out by applying a linear gradient of NaCl 0 - 1 M in the same buffer at a flow rate of 1 mL/min. The truncated form of MMP-8 was eluted at a salt concentration of 100 mM NaCl and could be purified to apparent homogeneity. A further step of purification was carried out by gel filtration using a Superdex 75 10/300 GL column (GE Healthcare) equilibrated with Tris 20 mM, pH 8.5, Urea 6 M, DTT 10 mM at a flow rate of 0.5 mL/min. The collected protein was then refolded onto a Superdex 75 10/300 GL column in buffer MES/NaOH 3 mM, pH 6.0, NaCl 100mM, $CaCl_2$ 5 mM, $ZnCl_2$ 0.5 mM, NaN_3 0.02% at a flow rate of 0.5 mL/min.

Protein Crystallization

The inhibitor (stock solution 50 mM in DMSO) was immediately added to the fraction containing the refolded protein in the ratio 3:1 (final concentration of DMSO 1%) in order to prevent autoproteolysis during concentration. The MMP-8 protein with the inhibitor was then concentrated with Amicon-Ultra-15 (Millipore), to a final concentration of 6 mg/mL. Crystallization was performed by hanging-drop vapor diffusion method at 20°C. Hanging droplets were made by mixing 2 μL of protein/inhibitor solution with 5 μL of PEG solution (PEG6000 10% w/v, MES/NaOH 0.2 M, pH 6.0, NaN_3 0.02%). Droplets were concentrated against a reservoir buffer containing Sodium Phosphate 1.0–2.0 M, pH 6.0, NaN_3 0.02%. Crystals appeared after few days.

Data Collection and Processing

X-ray data were collected under cryogenic conditions (100 K) at the ID29 beamline of ESRF, Grenoble, using a wavelength of 0.976 Å and a Pilatus 6M_F detector. The crystals were flash-frozen in the nitrogen stream after transferring them for few seconds into the mother solution containing 35% PEG400. Data were integrated and scaled using the programs MOSFLM and Scala.[1] The statistics of collection is given in Table S1.

Structure Solution and Refinement

Structure solution was performed with AMoRe[2] using the coordinates of the complex between MMP-8 and a non-zinc chelating inhibitor (PDB entry 3DPE)[3] as the starting model. The coordinates were then refined with CNS.[4] The statistics of refinement is summarized in Table S1.

Table S1. Statistics of crystallographic data and refinement for crystals of MMP-8 in complex with ML115.

	**ML115**
**Data collection**	
space group	$P2_12_12_1$
cell dimension *a*, *b*, *c* [Å]	32.91, 68.69, 70.69
wavelenght [Å]	0.976
resolution range [Å]	49.26 - 1.20
last shell [Å]	1.20 - 1.30
R_{merge} [%]	9.0 (45.6) [a]
unique reflections	45495
mean (*I*)/σ(*I*)	10.2 (2.5) [a]
completeness	99.2 (99.2) [a]
No. of molecules in asymmetric unit	1
**Refinement**	
resolution range [Å]	49.26 - 1.20
R_{work} [%]	17.0
R_{free} [%]	19.4
Bond lenghts r.m.s.d. [Å]	0.010
Bond angles r.m.s.d. [deg]	1.522
**PDB code**	4QKZ

[a] The values in parenthesis refer to the outer shell.

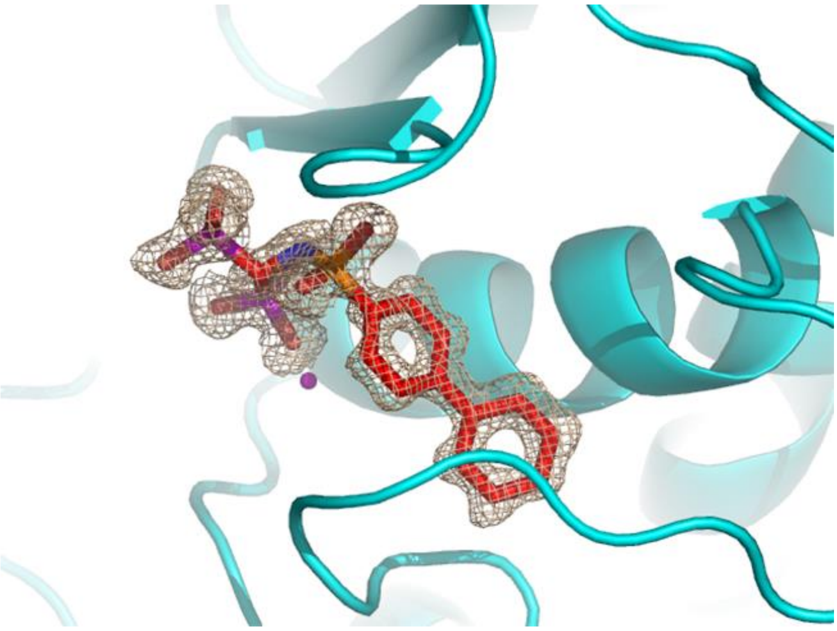

Figure S1. $2F_o$-F_c electron density map calculated around ML115 (red). The map is contoured at 1σ.

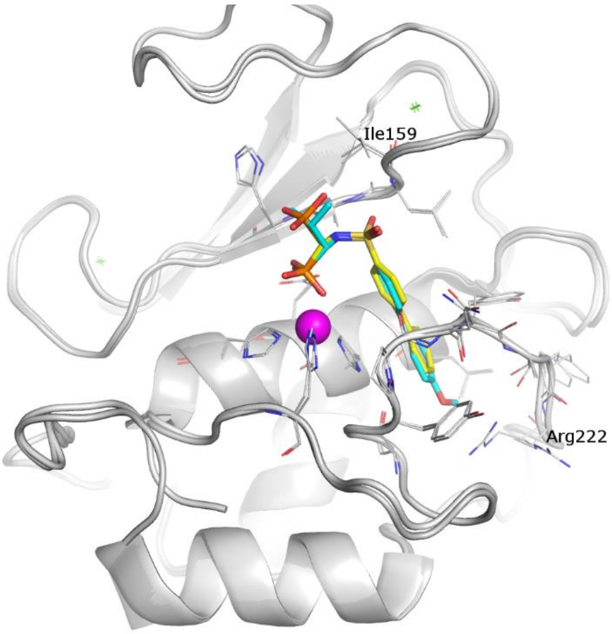

Figure S2. Superposition of the previously obtained X-ray complex MMP-8:monophosphonate sulfonamide (cyan C atoms) with the MMP-8:**ML 115** (yellow C atoms).

Proteins are represented as grey cartoon, the catalytic zinc ion is a magenta sphere, residues at 5 Å from the ligands are represented as thin sticks. Ligands and protein residues are almost perfectly superposed; main differences reside in the Ile159 and Arg222 positions.

Table S2. Enrichment values obtained ranking the docked compounds on the basis of calculated MM-GBSA DG bind. Compounds are considered active if their pIC_{50} ≥6.

	**MMP-2**	**MMP-8 (4KQZ)**	**MMP-8 (3DPF)**	**MMP-9**	**MMP-13**
BEDROC (alpha=160.9, alpha*Ra=34.4786)	1.000	0.000	1.000	1.000	1.000
BEDROC (alpha=20.0, alpha*Ra=4.2857)	0.815	0.011	0.990	0.966	0.956
BEDROC (alpha=8.0, alpha*Ra=1.7143)	0.705	0.120	0.921	0.926	0.840
ROC	0.79	0.53	0.90	0.97	0.85
RIE	3.75	0.04	3.45	4.45	4.40
AUC	0.73	0.52	0.79	0.87	0.77
Ave. Number of outranking decoys	2	4	1	0	1

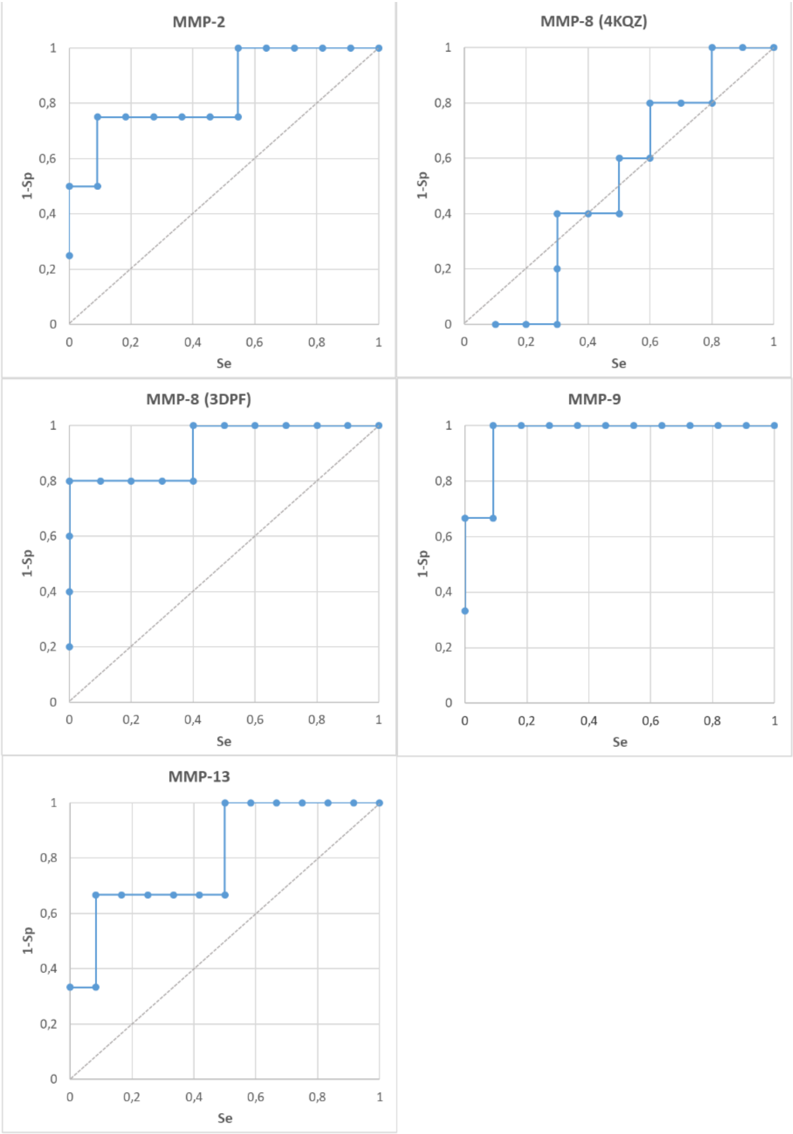

Figure S3. ROC curves obtained for inhibitor docked poses into MMP-2, MMP-8 (4KQZ), MMP-8 (3DPF), MMP-9 and MMP-13.

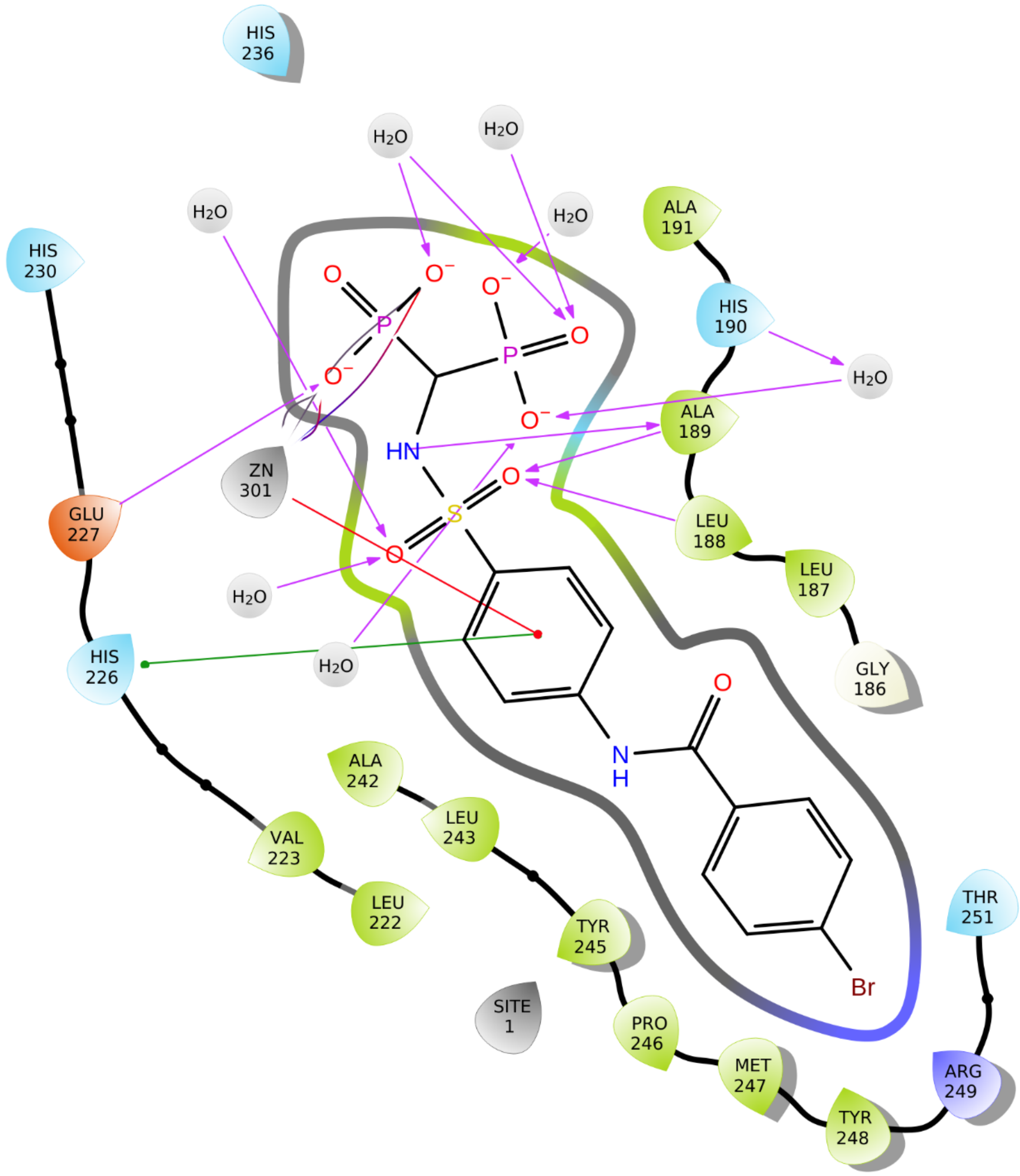

Figure S4. 2D ligand interaction diagram representing the contacts between ligand **9** docked in the MMP-9 binding site.

References

1. Battye, T. G. G.; Kontogiannis, L.; Johnson, O.; Powell, H. R.; Leslie, A. G. W. IMOSFLM: A New Graphical Interface for Diffraction-Image Processing with MOSFLM. Acta Crystallogr. Sect. D Biol. Crystallogr. 2011, 67 (4), 271–281. https://doi.org/10.1107/S0907444910048675.
2. Navaza, J. AMoRe: An Automated Package for Molecular Replacement. Acta Crystallogr. Sect. A 1994, 50 (2), 157–163. https://doi.org/10.1107/S0108767393007597.
3. Pochetti, G.; Montanari, R.; Gege, C.; Chevrier, C.; Taveras, A. G.; Mazza, F. Extra Binding Region Induced by Non-Zinc Chelating Inhibitors into the S 1 Subsite of Matrix Metalloproteinase 8 (MMP-8). J. Med. Chem. 2009, 52 (4), 1040–1049. https://doi.org/10.1021/jm801166j.
4. Brünger, A. T.; Adams, P. D.; Clore, G. M.; Delano, W. L.; Gros, P.; Grossekunstleve, R. W.; Jiang, J. S.; Kuszewski, J.; Nilges, M.; Pannu, N. S.; Read, R. J.; Rice, L. M.; Simonson, T.; Warren, G. L. Crystallography

& NMR System: A New Software Suite for Macromolecular Structure Determination. Acta Crystallogr. Sect. D Biol. Crystallogr. 1998, 54 (5), 905–921. https://doi.org/10.1107/S0907444998003254.

© 2020 by the authors. Licensee MDPI, Basel, Switzerland. This article is an open access article distributed under the terms and conditions of the Creative Commons Attribution (CC BY) license (http://creativecommons.org/licenses/by/4.0/).
